# Supplementary material for: Nutrition, Physical Activity and Smoking Habit in the Italian General Adult Population: CUORE Project Health Examination Survey 2018–2019
Source: Healthcare (Basel). 2024 Feb 15;12(4):475. doi: 10.3390/healthcare12040475 (PMC10888300; doi:10.3390/healthcare12040475)
Supplement: Supplementary file 1 [file healthcare-12-00475-s001.zip › SUPP_FIGURES_23_10_2023.pdf]

Figure S.1. Age-standardized (Italian population) type of daily consumed cigarettes by age classes, geographical area and educational level. Men residing in Italy aged 35-74 years, Health Examination Survey 2018-2019 - CUORE Project.

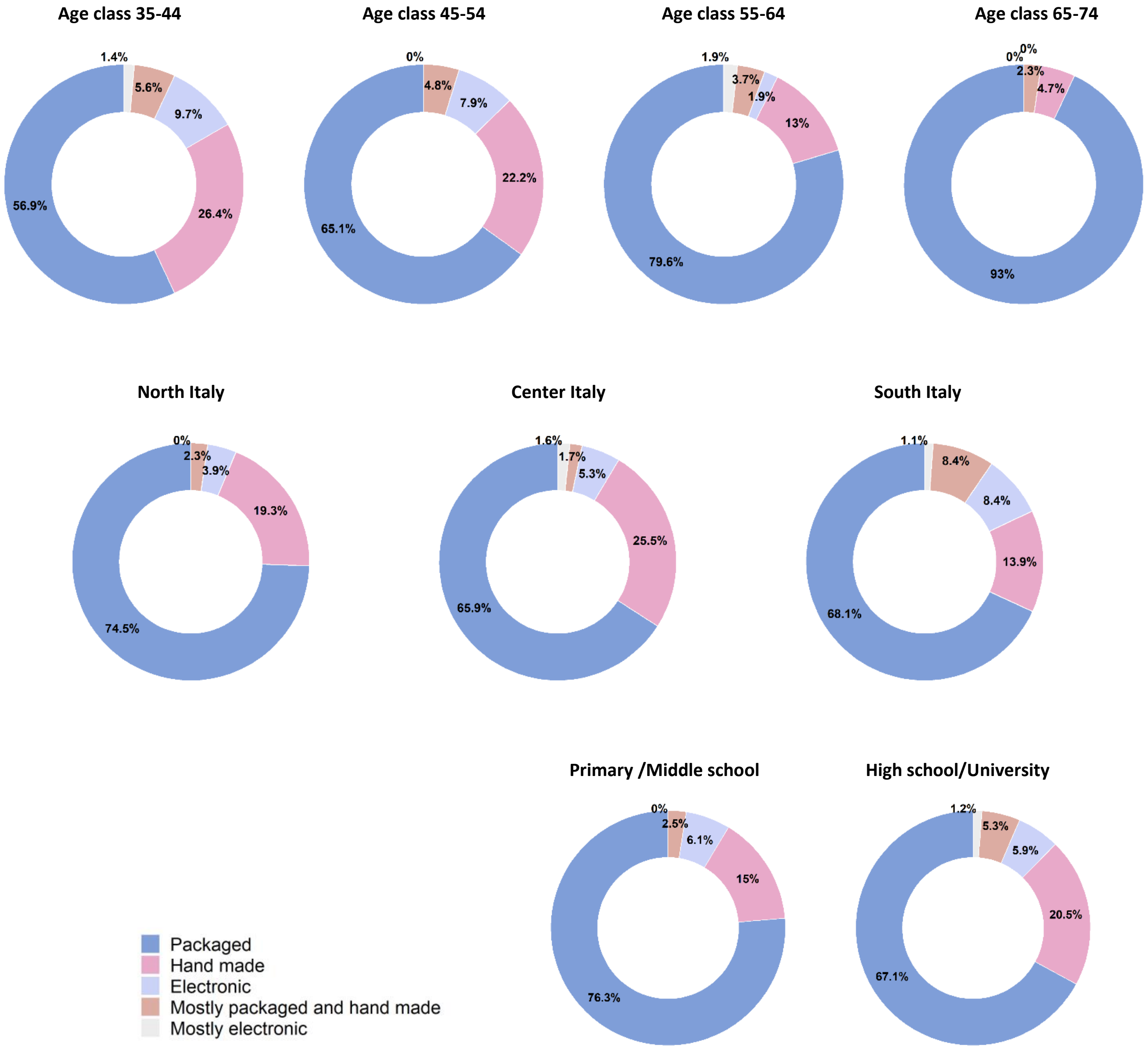

Pool of the following Italian regions: Piedmont, Lombardy, Liguria, Emilia Romagna, Tuscany, Lazio, Abruzzo, Basilicata, Calabria, and Sicily.
Prevalence was age standardized by Italian National Institute of Statistics-ISTAT Italian population 2019 (except when it is reported by age-classes).

Figure S.2. Age-standardized (Italian population) type of daily consumed cigarettes by age classes, geographical area and educational level. Women residing in Italy aged 35-74 years, Health Examination Survey 2018-2019 - CUORE Project.

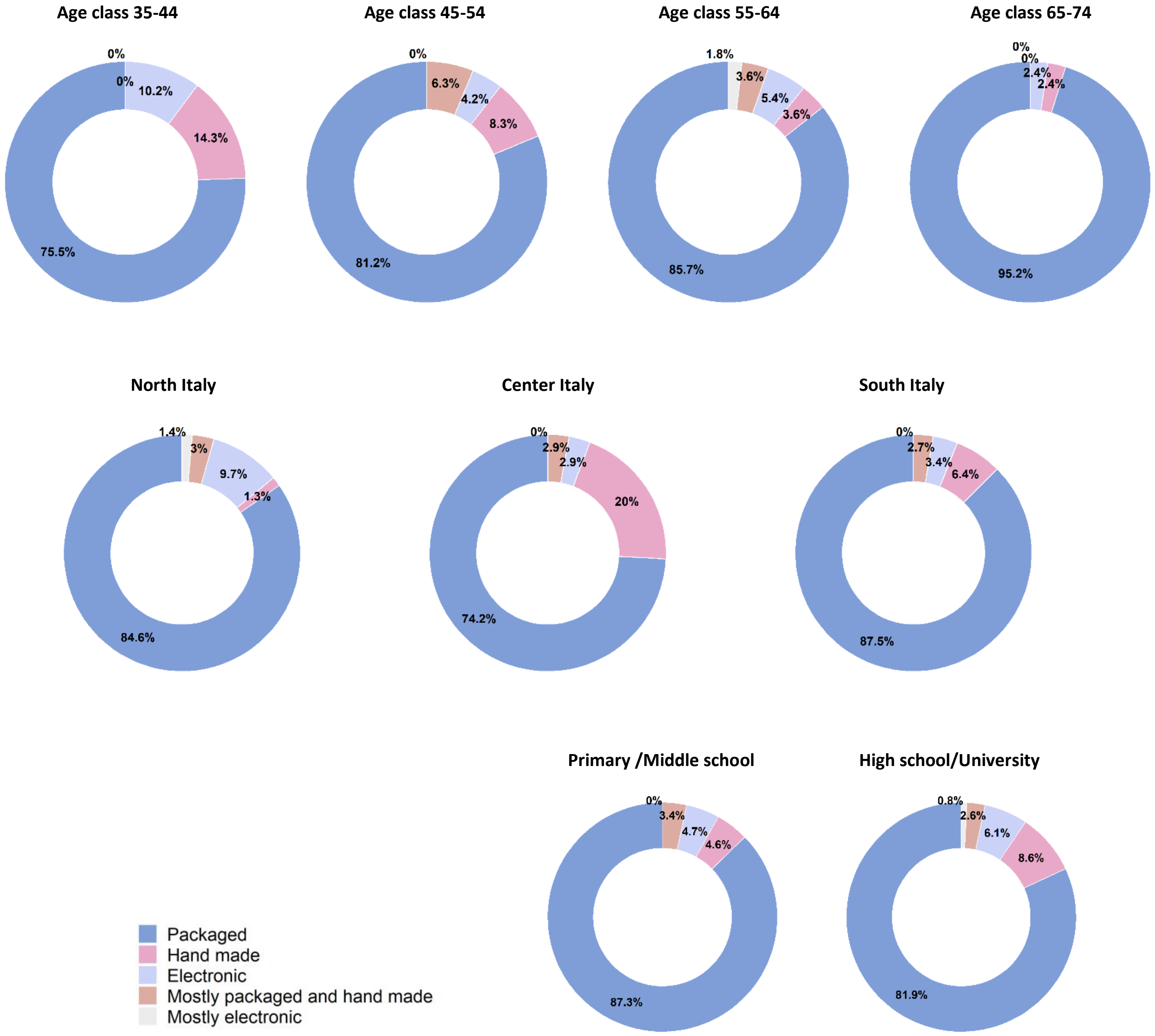

Pool of the following Italian regions: Piedmont, Lombardy, Liguria, Emilia Romagna, Tuscany, Lazio, Abruzzo, Basilicata, Calabria, and Sicily.
Prevalence was age standardized by Italian National Institute of Statistics-ISTAT Italian population 2019 (except when it is reported by age-classes).

Figure S.3. Age-standardized (Italian population) physical activity during leisure time by age classes, geographical area and educational level. Men residing in Italy aged 35-74 years, Health Examination Survey 2018-2019 - CUORE Project.

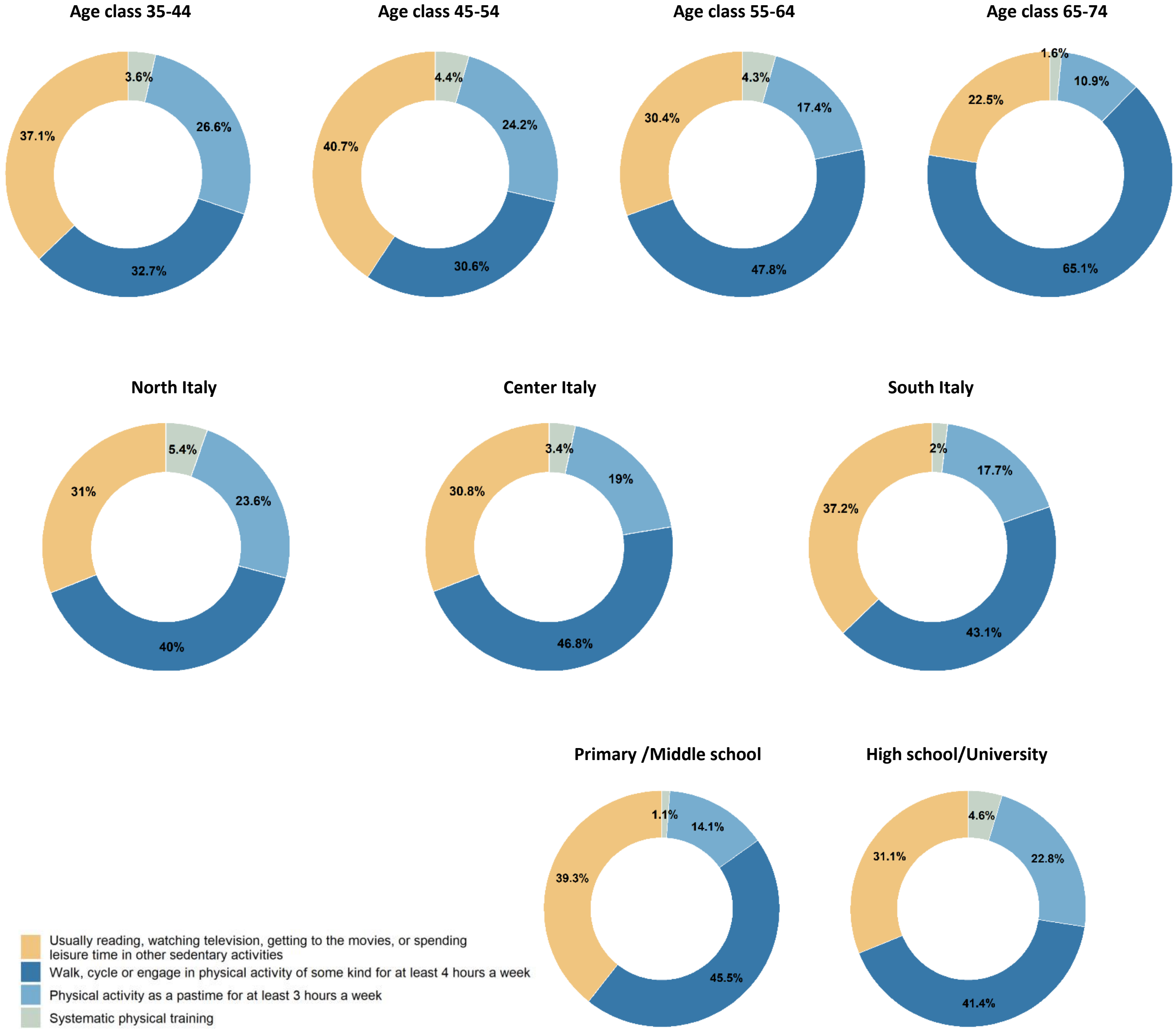

Pool of the following Italian regions: Piedmont, Lombardy, Liguria, Emilia Romagna, Tuscany, Lazio, Abruzzo, Basilicata, Calabria, and Sicily.
Prevalence was age standardized by Italian National Institute of Statistics-ISTAT Italian population 2019 (except when it is reported by age-classes).

Figure S.4. Age-standardized (Italian population) physical activity during leisure time by age classes, geographical area and educational level. Women residing in Italy aged 35-74 years, Health Examination Survey 2018-2019 - CUORE Project.

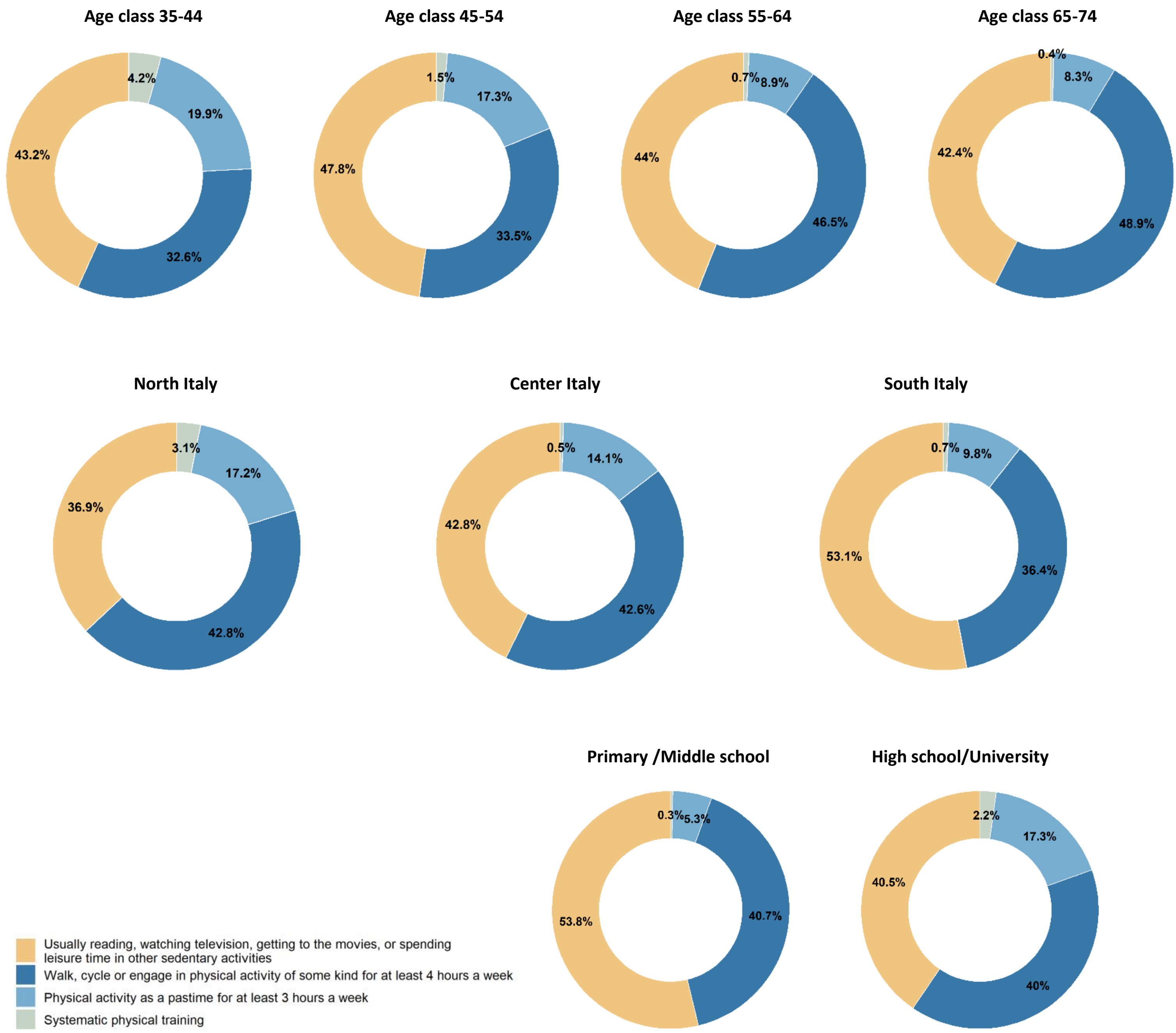

Pool of the following Italian regions: Piedmont, Lombardy, Liguria, Emilia Romagna, Tuscany, Lazio, Abruzzo, Basilicata, Calabria, and Sicily.
Prevalence was age standardized by Italian National Institute of Statistics-ISTAT Italian population 2019 (except when it is reported by age-classes).

Figure S.5. Age-standardized (Italian population) physical activity at work by age classes, geographical area and educational level. Men residing in Italy aged 35-74 years, Health Examination Survey 2018-2019 - CUORE Project.

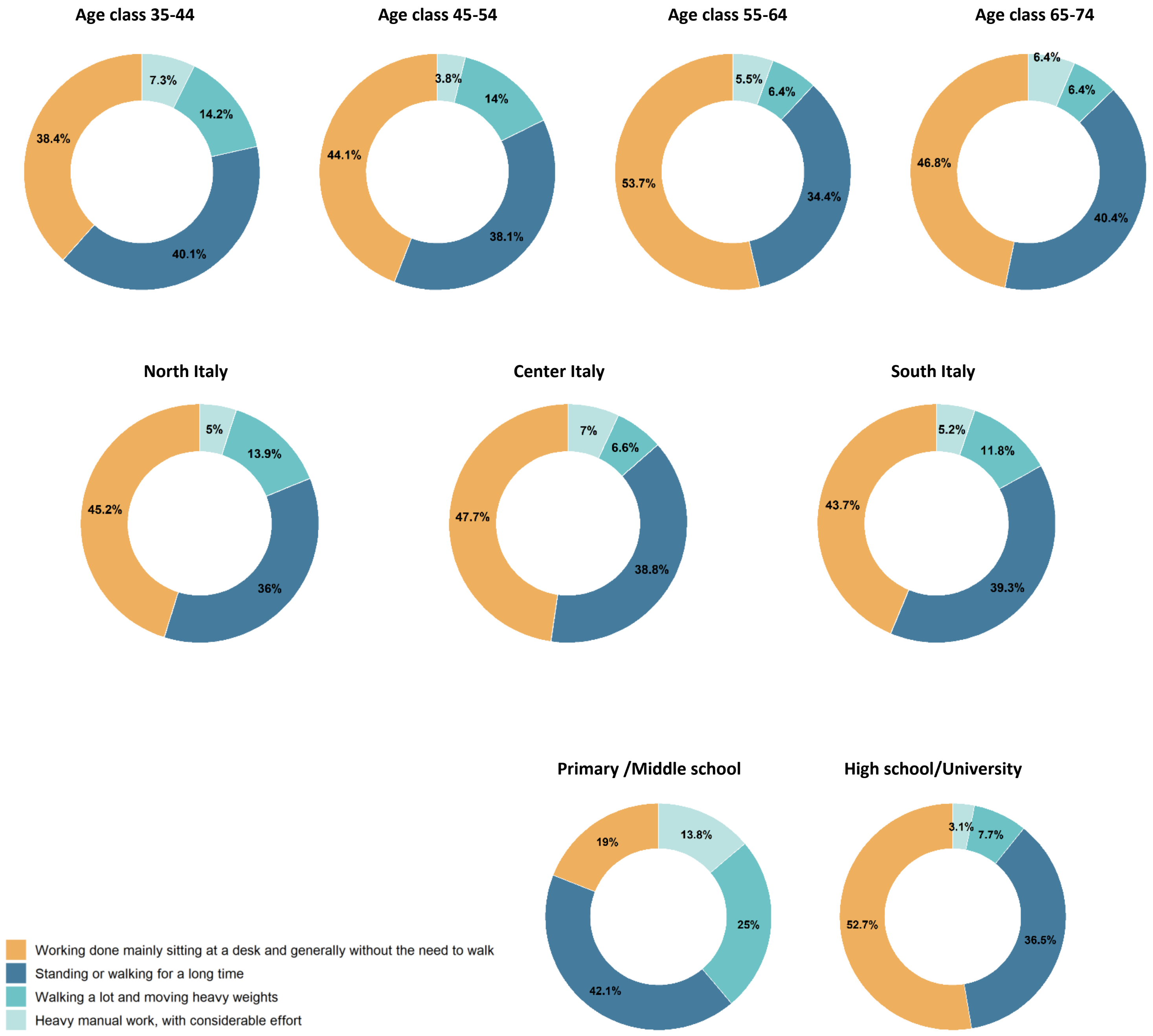

Pool of the following Italian regions: Piedmont, Lombardy, Liguria, Emilia Romagna, Tuscany, Lazio, Abruzzo, Basilicata, Calabria, and Sicily.
Prevalence was age standardized by Italian National Institute of Statistics-ISTAT Italian population 2019 (except when it is reported by age-classes).

Figure S.6. Age-standardized (Italian population) physical activity at work by age classes, geographical area and educational level. Women residing in Italy aged 35-74 years, Health Examination Survey 2018-2019 - CUORE Project.

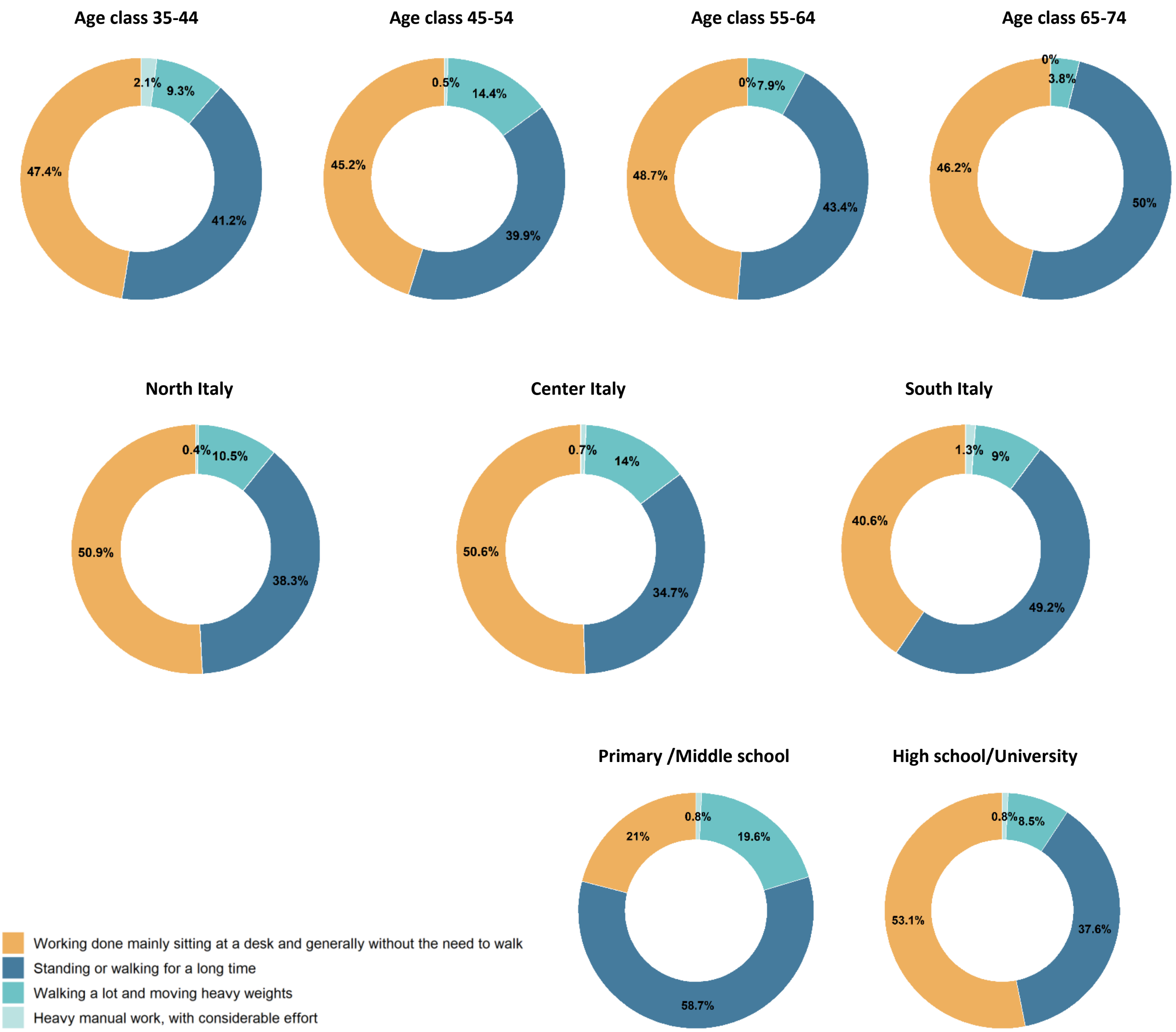

Pool of the following Italian regions: Piedmont, Lombardy, Liguria, Emilia Romagna, Tuscany, Lazio, Abruzzo, Basilicata, Calabria, and Sicily.
Prevalence was age standardized by Italian National Institute of Statistics-ISTAT Italian population 2019 (except when it is reported by age-classes).
