# Supplementary material for: Nutrition, Physical Activity and Smoking Habit in the Italian General Adult Population: CUORE Project Health Examination Survey 2018–2019
Source: Healthcare (Basel). 2024 Feb 15;12(4):475. doi: 10.3390/healthcare12040475 (PMC10888300; doi:10.3390/healthcare12040475)
Supplement: Supplementary file 1 [file healthcare-12-00475-s001.zip › SUPP_TABLES_23_10_2023.pdf]

Table S.1. Age-standardized (Italian population) current smokers and former smokers prevalence and mean of number of cigarettes smoked per day by region.

Men and women residing in Italy aged 35-74 years, Health Examination Survey 2018-2019 - CUORE Project.

	Men						Women					
**Current Smokers**												
	N	n	%	CI 95%		χ^2 p-value	N	n	%	CI 95%		χ^2 p-value
**Italian Region**												
Abruzzo	103	24	24,2	7,5	40,8	0,1115	106	24	22,6	6,6	38,6	0,8172
Basilicata	106	24	22,4	6,4	38,5		113	16	14,2	1,3	27,1	
Calabria	102	18	17,8	2,9	32,8		108	11	10,8	0,0	22,5	
Emilia-Romagna	103	22	21,2	5,4	37,0		105	19	18,0	3,2	32,9	
Lazio	99	30	31,2	12,9	49,4		114	24	21,3	6,1	36,5	
Liguria	104	20	19,0	3,8	34,1		107	18	17,0	2,8	31,3	
Lombardia	98	22	23,7	6,7	40,6		99	18	18,1	2,7	33,4	
Piemonte	104	24	22,5	6,3	38,7		110	19	16,9	2,9	31,0	
Sicilia	105	22	21,9	5,9	37,9		103	31	30,4	12,5	48,3	
Toscana	107	28	27,7	10,7	44,7		94	16	17,3	2,0	32,6	
**Daily smoke - number of cigarettes**												
	N	Mean	SD	CI 95%		anova p-value	N	Mean	SD	CI 95%		anova p-value
**Italian Region**												
Abruzzo	24	11,0	8,2	9,4	12,5	0,0090	24	9,6	4,9	8,7	10,6	0,0090
Basilicata	24	13,9	9,8	12,1	15,8		16	10,0	5,2	9,0	11,0	
Calabria	18	17,4	8,4	15,8	19,1		11	8,9	6,0	7,8	10,1	
Emilia-Romagna	22	14,7	11,6	12,5	17,0		19	15,1	9,6	13,3	17,0	
Lazio	30	14,6	8,2	13,0	16,2		24	10,6	5,9	9,5	11,7	
Liguria	20	12,1	7,2	10,7	13,5		18	10,1	7,4	8,7	11,5	
Lombardia	22	14,7	10,3	12,7	16,7		18	8,5	5,4	7,4	9,5	
Piemonte	24	9,7	9,2	7,9	11,5		19	9,0	6,8	7,7	10,2	
Sicilia	22	18,1	5,8	17,0	19,2		31	12,8	5,3	11,8	13,8	
Toscana	28	9,7	6,0	8,6	10,9		16	11,0	5,4	9,9	12,1	
**Former Smokers**												
	N	n	%	CI 95%		χ^2 p-value	N	n	%	CI 95%		χ^2 p-value
**Italian Region**												
Abruzzo	103	37	44,8	25,4	64,1	0,1116	106	25	29,7	12,2	47,2	0,0158
Basilicata	106	43	50,3	31,0	69,6		113	24	24,2	8,3	40,0	
Calabria	102	33	37,8	18,9	56,7		108	16	16,5	2,5	30,5	
Emilia-Romagna	103	39	47,7	28,4	67,1		105	31	36,5	17,9	55,2	
Lazio	99	30	42,9	23,3	62,5		114	28	30,3	13,2	47,4	
Liguria	104	28	33,2	15,0	51,4		107	21	23,5	7,4	39,5	
Lombardia	98	30	37,8	18,4	57,1		99	20	23,4	6,5	40,3	
Piemonte	104	33	40,9	21,8	59,9		110	33	35,0	17,1	52,9	
Sicilia	105	47	54,4	35,1	73,7		103	17	22,5	6,2	38,7	
Toscana	107	31	39,1	20,5	57,7		94	31	38,1	18,4	57,7	

N: number of participants denominator.
n: number of participants related to the prevalence numerator.
Current smoker is defined as a person who smokes one or more cigarettes per day.
A former smoker is defined as a person who has quit smoking for at least 12 months.
Number of cigarettes refers to packaged, handmade or electronic cigarettes.
SD: standard deviation; CI: confidence interval.
Means, standard deviations, and prevalence were age standardized by Italian National Institute of Statistics-ISTAT Italian population 2019 (except when they are reported by age-classes).
Anova to compare mean values among classes; chi-square test to compare prevalence among classes.

Table S.2. Age-standardized (Italian population) sedentariness during leisure time and at work prevalence by region. Men and women residing in Italy aged 35-74 years, Health Examination Survey 2018-2019 - CUORE Project.

	Men						Women					
**Sedentariness during leisure time**												
	N	n	%	CI 95%		χ^2 p-value	N	n	%	CI 95%		χ^2 p-value
**Italian Region**												
Abruzzo	105	32	30,9	13,1	48,7	0,1783	106	52	49,2	30,1	68,3	<0.0001
Basilicata	106	29	29,4	11,8	47,0		114	56	49,8	31,5	68,2	
Calabria	102	48	49,0	29,6	68,5		109	52	48,1	29,4	66,8	
Emilia-Romagna	104	47	46,6	27,5	65,8		106	47	44,6	25,5	63,7	
Lazio	99	33	35,0	16,1	53,8		115	55	48,1	29,6	66,5	
Liguria	104	28	27,2	10,1	44,4		107	33	31,6	14,0	49,3	
Lombardia	98	27	28,5	10,5	46,5		100	42	42,6	23,0	62,2	
Piemonte	104	22	21,5	5,6	37,4		113	33	30,1	13,1	47,0	
Sicilia	105	40	39,7	20,7	58,6		103	68	65,8	47,4	84,3	
Toscana	108	29	27,1	10,2	43,9		98	36	36,8	17,7	55,9	
**Sedentariness at work**												
	N	n	%	CI 95%		χ^2 p-value	N	n	%	CI 95%		χ^2 p-value
**Italian Region**												
Abruzzo	82	33	44,2	23,0	65,4	0,0023	85	31	42,6	21,7	63,4	0,0595
Basilicata	76	36	50,0	27,9	72,0		87	30	45,7	25,1	66,4	
Calabria	71	27	41,7	19,2	64,2		93	14	29,4	11,1	47,8	
Emilia-Romagna	79	27	35,0	14,5	55,4		75	23	38,0	16,1	59,9	
Lazio	76	35	47,6	25,7	69,6		88	29	47,2	26,4	68,1	
Liguria	83	39	48,4	27,2	69,6		81	32	50,4	28,9	71,9	
Lombardia	68	19	29,1	8,0	50,1		71	25	49,6	26,4	72,8	
Piemonte	85	55	64,3	44,1	84,5		87	48	61,6	41,5	81,8	
Sicilia	64	23	38,1	14,8	61,5		88	21	41,6	20,9	62,3	
Toscana	83	38	47,7	26,6	68,9		78	37	53,6	31,8	75,4	
**Sedentariness during leisure time and at work**												
	N	n	%	CI 95%		χ^2 p-value	N	n	%	CI 95%		χ^2 p-value
**Italian Region**												
Abruzzo	78	8	10,8	0,0	24,1	0,2512	77	16	20,3	3,3	37,2	0,8029
Basilicata	74	11	15,2	0,0	31,1		77	15	19,9	3,4	36,4	
Calabria	70	18	26,6	6,4	46,8		73	11	15,0	0,7	29,3	
Emilia-Romagna	78	12	15,9	0,0	31,6		66	10	15,6	0,0	31,9	
Lazio	74	14	19,4	2,0	36,7		76	11	14,8	0,0	29,6	
Liguria	82	10	12,2	0,0	26,0		77	10	12,8	0,0	27,2	
Lombardia	67	7	10,3	0,0	24,5		68	13	19,8	1,4	38,3	
Piemonte	85	12	14,9	0,0	29,9		85	16	19,9	3,4	36,4	
Sicilia	63	12	19,6	0,5	38,6		60	12	19,8	3,1	36,5	
Toscana	81	10	12,2	0,0	26,1		75	18	23,7	5,1	42,3	

N: number of participants denominator.
n: number of participants related to the prevalence numerator.
Sedentariness during leisure time is defined as someone who usually carries out activities such as reading, watching television, getting to the movies or spend leisure time in other sedentary activities.
Sedentariness at work is defined as one whose work mainly involves sitting at a desk and generally without the need to walk.
SD: standard deviation; CI: confidence interval.
Prevalence was age standardized by Italian National Institute of Statistics-ISTAT Italian population 2019 (except when it is reported by age-classes).
Chi-square test to compare prevalence among classes.

Table S.3. Age-standardized (Italian population) sedentary retirees prevalence by sex, age classes, geographical area and educational level.

Men and women residing in Italy aged 35-74 years, Health Examination Survey 2018-2019 - CUORE Project.

	Men						Women					
	% Sedentariness during leisure time among retirees											
	N	n	%	CI 95%		χ^2 p-value	N	n	%	CI 95%		χ^2 p-value
**All**	**268**	**101**	**37,8**	**31,9**	**43,7**		**238**	**125**	**51,9**	**45,9**	**57,9**	
**Age classes (years)**												
35-44	9	6	66,7	35,9	97,5	0,2658	7	1	14,3	0,0	40,2	0,0506
45-54	5	1	20,0	0,0	55,1		7	2	28,6	0,0	62,0	
55-64	50	19	38,0	24,6	51,5		38	17	44,7	28,9	60,6	
65-74	204	75	36,8	30,2	43,4		186	105	56,5	49,3	63,6	
**Italian Area**												
North	95	36	38,6	29,2	48,1	0,2198	112	57	50,2	40,6	59,7	0,7872
Center	48	23	48,5	34,8	62,3		47	24	50,9	37,3	64,5	
South	125	42	33,2	24,1	42,3		79	44	54,9	45,5	64,3	
**Italian Region**												
Abruzzo	23	8	35,7	16,9	54,5	0,2658	21	13	60,9	41,9	79,9	0,0506
Basilicata	30	13	43,1	23,8	62,5		27	16	58,6	40,2	77,0	
Calabria	31	10	31,2	12,9	49,5		16	8	50,6	31,4	69,9	
Emilia-Romagna	25	9	37,6	18,7	56,5		31	11	35,4	16,8	54,0	
Lazio	23	11	48,4	28,4	68,5		27	12	45,2	26,5	63,8	
Liguria	21	9	43,9	24,3	63,4		26	9	34,7	16,3	53,0	
Lombardia	30	7	22,2	5,4	39,0		29	18	60,3	40,6	79,9	
Piemonte	19	11	60,1	40,7	79,5		26	19	72,7	56,0	89,4	
Sicilia	25	12	48,6	29,4	67,9		20	12	58,6	38,7	78,5	
Toscana	41	11	26,5	9,3	43,7		15	7	44,6	24,7	64,5	
**Education**												
Primary and middle school	129	28	21,6	12,1	31,1	<0,0001	110	34	30,9	20,8	40,9	<0,0001
High school and university	139	73	52,7	45,5	60,0		128	91	69,6	62,9	76,2	

N: number of participants denominator.
n: number of participants related to the prevalence numerator.
Retired: participants who no longer carries out a work activity.
SD: standard deviation; CI: confidence interval.
Prevalence was age standardized by Italian National Institute of Statistics-ISTAT Italian population 2019 (except when it is reported by age-classes).
Chi-square test to compare prevalence among classes.

Table S.4. Age-standardized (Italian population) balanced nutrition food groups intake (EPIC questionnaire): vegetables, fruit, fish and cheese prevalence by region.
Men and women residing in Italy aged 35-74 years, Health Examination Survey 2018-2019 - CUORE Project.

	Men						Women					
**Vegetables**												
	N	n	%	CI 95%		χ^2 p-value	N	n	%	CI 95%		χ^2 p-value
Italian Region												
Basilicata	88	25	28,8	9,8	47,8	0,2081	95	36	37,4	18,0	56,8	0,6029
Calabria	75	24	31,8	10,9	52,7		67	24	35,6	13,0	58,1	
Emilia-Romagna	86	21	24,3	6,4	42,3		81	32	40,5	19,0	61,9	
Lazio	82	28	33,5	12,9	54,2		102	40	40,0	20,8	59,2	
Liguria	98	26	27,5	9,8	45,3		100	42	42,3	23,0	61,6	
Lombardia	80	18	22,4	4,0	40,7		81	27	33,5	12,8	54,2	
Piemonte	93	32	34,3	14,9	53,7		97	39	40,6	21,0	60,1	
Sicilia	93	17	19,0	3,0	35,1		80	24	30,0	9,8	50,1	
Toscana	98	31	32,5	13,9	51,0		82	38	47,1	25,6	68,6	
**Fruit**												
	N	n	%	CI 95%		χ^2 p-value	N	n	%	CI 95%		χ^2 p-value
Italian Region												
Basilicata	88	42	49,0	28,0	70,0	0,0745	95	51	52,8	32,7	72,8	0,5315
Calabria	75	25	33,7	12,5	54,9		67	28	41,5	18,3	64,7	
Emilia-Romagna	86	38	43,6	22,9	64,4		81	50	62,3	41,1	83,5	
Lazio	82	45	53,9	32,1	75,7		102	54	52,4	32,9	71,9	
Liguria	98	47	48,3	28,5	68,2		100	49	50,2	30,6	69,7	
Lombardia	80	47	58,4	36,7	80,1		81	43	53,7	31,8	75,5	
Piemonte	93	47	50,5	30,0	70,9		97	54	56,3	36,5	76,1	
Sicilia	93	51	55,5	35,1	75,8		80	40	48,9	26,9	70,9	
Toscana	98	52	53,2	33,4	73,0		82	42	50,9	29,4	72,5	
**Vegetable and Fruit**												
	N	n	%	CI 95%		χ^2 p-value	N	n	%	CI 95%		χ^2 p-value
Italian Region												
Basilicata	88	10	12,3	0,0	26,1	<0,0001	95	17	17,4	2,2	32,6	<0,0001
Calabria	75	4	5,7	0,0	16,1		67	7	10,4	0,0	24,7	
Emilia-Romagna	86	9	9,9	0,0	22,4		81	22	27,7	8,1	47,3	
Lazio	82	15	18,4	1,5	35,3		102	15	15,0	1,0	29,0	
Liguria	98	13	13,8	0,1	27,5		100	22	22,7	6,3	39,0	
Lombardia	80	11	14,4	0,0	29,9		81	15	19,1	1,9	36,4	
Piemonte	93	17	18,4	2,5	34,2		97	23	24,1	7,0	41,1	
Sicilia	93	10	11,5	0,0	24,5		80	15	18,9	1,7	36,2	
Toscana	98	15	15,8	1,4	30,3		82	18	22,6	4,6	40,6	
**Fish**												
	N	n	%	CI 95%		χ^2 p-value	N	n	%	CI 95%		χ^2 p-value
Italian Region												
Basilicata	88	38	43,6	22,8	64,4	0,0063	95	42	44,1	24,2	64,0	0,5027
Calabria	75	36	47,3	24,9	69,6		67	26	38,5	15,6	61,4	
Emilia-Romagna	86	47	55,9	35,1	76,7		81	36	43,9	22,2	65,7	
Lazio	82	44	53,7	31,9	75,5		102	42	40,6	21,4	59,8	
Liguria	98	49	50,1	30,3	70,0		100	48	47,1	27,6	66,6	
Lombardia	80	25	32,4	11,8	53,0		81	28	33,3	12,6	54,0	
Piemonte	93	44	47,2	26,8	67,7		97	32	33,3	14,5	52,0	
Sicilia	93	35	38,2	18,3	58,1		80	34	43,5	21,7	65,3	
Toscana	98	32	31,9	13,4	50,3		82	31	38,5	17,6	59,5	
**Cheese**												
	N	n	%	CI 95%		χ^2 p-value	N	n	%	CI 95%		χ^2 p-value
Italian Region												
Basilicata	88	41	46,4	25,5	67,4	0,6374	95	55	58,8	39,0	78,5	0,4995
Calabria	75	42	56,1	33,8	78,3		67	30	44,1	20,7	67,5	
Emilia-Romagna	86	34	39,9	19,4	60,4		81	40	49,8	27,9	71,7	
Lazio	82	37	45,1	23,4	66,8		102	52	50,6	31,0	70,2	
Liguria	98	43	43,4	23,8	63,1		100	56	56,6	37,3	76,0	
Lombardia	80	32	40,3	18,7	61,9		81	42	51,6	29,7	73,5	
Piemonte	93	40	42,2	22,0	62,5		97	51	52,2	32,3	72,1	
Sicilia	93	40	43,2	22,9	63,5		80	51	63,9	42,8	85,0	
Toscana	98	46	46,0	26,2	65,8		82	44	53,9	32,4	75,4	

N: number of participants denominator.
n: number of participants related to the prevalence numerator.
For vegetables intake, the following foods were considered: leafy vegetables-cooked, leafy vegetables-raw, other vegetables, tomatoes-raw, tomatoes-cooked, root vegetables, cabbages, mushrooms, grain and pod vegetables, onion, garlic, stalk vegetables, mixed salad, mixed vegetables.
For vegetables intake were considered the following foods: leafy vegetable-cooked, leafy vegetable-raw, other vegetables, tomatoes-raw, tomatoes-cooked, root vegetables, cabbages, mushrooms, grain and pod vegetables, onion, garlic, stalk vegetables, mixed salad, mixed vegetables.
For fruits intake, the following foods were considered: citrus fruits and other fruits.
For fish intake, the following foods were considered: fish, crustaceans and molluscs.
For cheese intake, the following foods were considered: cheeses (including fresh cheeses).
Dietary information was collected by the self-administered Italian version of the food frequency questionnaire (FFQ) of the European Prospective Investigation into Cancer (EPIC).
A balanced nutrition was defined based on the following intake: vegetables ≥200 g/day; fruit 150-375 g/day; fish at least twice per week (150 g per serving); cheese no more than three times per week (50-100 g per serving); sausages, salami and other preserved meat no more than once per week (50 g per serving); cake and desserts no more than once per week (100 g per serving); sugar beverages less than one per week (330 ml); and consumption of alcoholic beverages limited to two glasses per day for men (24 g of ethanol), one glass per day for women (12 g of ethanol) and one glass per day for men and women aged 65-74 (12 g of ethanol).
SD: standard deviation; CI: confidence interval.

Prevalence was age standardized by Italian National Institute of Statistics-ISTAT Italian population 2019 (except when it is reported by age-classes).
Chi-square test to compare prevalence among classes.

Table S.5. Age-standardized (Italian population) balanced nutrition food groups intake (EPIC questionnaire): processed meat, sweets/cakes, sweet drinks and alcohol prevalence by region. Men and women residing in Italy aged 35-74 years, Health Examination Survey 2018-2019 - CUORE Project.

	Men						Women					
**Processed meat**												
	N	n	%	CI 95%		χ^2 p-value	N	n	%	CI 95%		χ^2 p-value
Italian Region												
Basilicata	88	6	6,8	0,0	17,4	0,2917	95	19	19,3	3,5	35,2	0,0975
Calabria	75	9	11,9	0,0	26,3		67	12	17,7	0,0	35,6	
Emilia-Romagna	86	4	4,5	0,0	13,2		81	13	15,5	0,0	31,3	
Lazio	82	11	12,7	0,0	27,3		102	17	16,3	1,8	30,7	
Liguria	98	12	12,1	0,0	25,1		100	24	23,8	7,2	40,5	
Lombardia	80	10	11,7	0,0	25,8		81	15	17,7	1,0	34,4	
Piemonte	93	11	11,8	0,0	25,1		97	31	30,7	12,3	49,1	
Sicilia	93	11	11,6	0,0	24,7		80	14	17,2	0,6	33,8	
Toscana	98	17	17,0	2,1	31,9		82	23	27,7	8,4	47,0	
**Sweets/Cakes**												
	N	n	%	CI 95%		χ^2 p-value	N	n	%	CI 95%		χ^2 p-value
Italian Region												
Basilicata	88	7	8,4	0,0	20,0	0,1054	95	7	8,0	0,0	18,9	0,2031
Calabria	75	15	18,8	1,3	36,3		67	12	18,2	0,0	36,4	
Emilia-Romagna	86	5	5,6	0,0	15,3		81	11	12,6	0,0	27,1	
Lazio	82	6	7,3	0,0	18,7		102	11	10,0	0,0	21,7	
Liguria	98	10	10,4	0,0	22,4		100	11	11,0	0,0	23,3	
Lombardia	80	7	7,9	0,0	19,8		81	6	6,8	0,0	17,9	
Piemonte	93	7	7,6	0,0	18,4		97	9	8,9	0,0	20,3	
Sicilia	93	10	9,4	0,0	21,4		80	14	17,0	0,5	33,5	
Toscana	98	7	6,7	0,0	16,5		82	6	7,2	0,0	18,3	
**Sweet Drinks**												
	N	n	%	CI 95%		χ^2 p-value	N	n	%	CI 95%		χ^2 p-value
Italian Region												
Basilicata	88	44	49,9	28,9	70,9	0,6748	95	59	62,0	42,5	81,5	0,5949
Calabria	75	39	50,0	27,6	72,4		67	40	59,8	36,7	82,9	
Emilia-Romagna	86	50	56,4	35,7	77,2		81	44	53,0	31,1	74,8	
Lazio	82	43	52,2	30,3	74,0		102	54	52,6	33,0	72,1	
Liguria	98	52	52,5	32,6	72,3		100	58	58,0	38,8	77,3	
Lombardia	80	44	54,7	32,8	76,7		81	50	60,0	38,5	81,5	
Piemonte	93	48	51,7	31,2	72,1		97	64	65,2	46,2	84,2	
Sicilia	93	60	64,2	44,6	83,9		80	50	63,1	41,8	84,3	
Toscana	98	52	53,3	33,5	73,1		82	54	65,0	44,5	85,6	
**Alcohol**												
	N	n	%	CI 95%		χ^2 p-value	N	n	%	CI 95%		χ^2 p-value
Italian Region												
Basilicata	88	56	65,4	45,4	85,4	0,0320	95	81	84,9	70,5	99,3	0,0320
Calabria	75	58	77,5	58,8	96,2		67	63	93,6	82,1	105,1	
Emilia-Romagna	86	57	66,7	47,0	86,5		81	68	84,6	68,8	100,4	
Lazio	82	69	85,6	70,3	101,0		102	86	83,7	69,2	98,1	
Liguria	98	70	72,5	54,8	90,2		100	80	80,4	64,9	95,9	
Lombardia	80	57	70,9	50,9	90,9		81	69	84,9	69,2	100,6	
Piemonte	93	64	70,8	52,2	89,4		97	75	77,3	60,6	94,0	
Sicilia	93	76	82,6	67,0	98,1		80	73	91,3	78,9	103,7	
Toscana	98	74	76,7	59,9	93,5		82	67	82,1	65,5	98,6	

N: number of participants denominator.
n: number of participants related to the prevalence numerator.
For processed meat intake, the following foods were considered: sausages, salami and other preserved meat.
For sweets/cakes intake, the following foods were considered: sugar, honey, jam, chocolate, candy bars, paste, confetti/flakes, confectionery non chocolate, ice cream, cakes, pies, pastries, puddings (not milk based), dry cakes, biscuits.
For sweet beverages intake, the following foods were considered: fruit and vegetable juices, carbonated/soft/isotonic drinks, diluted syrups.
For alcohol intake, the following foods were considered: alcoholic beverages.
Dietary information was collected by the self-administered Italian version of the food frequency questionnaire (FFQ) of the European Prospective Investigation into Cancer (EPIC).
A balanced nutrition was defined based on the following intake: vegetables ≥200 g/day; fruit 150-375 g/day; fish at least twice per week (150 g per serving); cheese no more than three times per week (50-100 g per serving); sausages, salami and other preserved meat no more than once per week (50 g per serving); cake and desserts no more than once per week (100 g per serving); sugar beverages less than one per week (330 ml); and consumption of alcoholic beverages limited to two glasses per day for men (24 g of ethanol), one glass per day for women (12 g of ethanol) and one glass per day for men and women aged 65-74 (12 g of ethanol).
SD: standard deviation; CI: confidence interval.
Prevalence was age standardized by Italian National Institute of Statistics-ISTAT Italian population 2019 (except when it is reported by age-classes).
Chi-square test to compare prevalence among classes.

Table S.6. Age-standardized (Italian population) balanced nutrition (EPIC questionnaire): vegetables, fruit, fish and cheese mean by region.
Men and women residing in Italy aged 35-74 years, Health Examination Survey 2018-2019 - CUORE Project.

	Men						Women					
	**Vegetable (g/day)**											
	N	Mean	SD	CI 95%		anova p-value	N	Mean	SD	CI 95%		anova p-value
Italian Region												
Basilicata	88	161,4	109,1	138,6	184,2	<0,0001	95	181,2	85,0	164,1	198,3	<0,0001
Calabria	75	168,4	105,0	144,7	192,2		67	181,1	92,8	158,9	203,3	
Emilia-Romagna	86	169,2	95,1	149,1	189,3		81	185,5	99,2	163,9	207,1	
Lazio	82	183,1	88,0	164,1	202,2		102	191,5	89,8	174,0	208,9	
Liguria	98	169,7	86,2	152,7	186,8		100	175,9	105,9	155,1	196,6	
Lombardia	80	143,5	93,6	123,0	164,0		81	167,4	97,4	146,2	188,6	
Piemonte	93	183,1	89,8	164,8	201,3		97	203,6	93,9	184,9	222,3	
Sicilia	93	151,7	80,6	135,3	168,1		80	157,4	87,4	138,3	176,6	
Toscana	98	182,8	91,7	164,6	200,9		82	210,5	107,1	187,3	233,6	
	**Fruit (g/day)**											
	N	Mean	SD	CI 95%		anova p-value	N	Mean	SD	CI 95%		anova p-value
Italian Region												
Basilicata	88	272,6	175,3	235,9	309,2	0,0269	95	285,2	168,3	251,4	319,1	0,0269
Calabria	75	331,0	215,4	282,3	379,8		67	267,9	174,1	226,2	309,6	
Emilia-Romagna	86	279,0	197,7	237,2	320,8		81	299,0	141,6	268,2	329,8	
Lazio	82	282,3	175,9	244,2	320,4		102	286,9	167,1	254,5	319,3	
Liguria	98	264,5	155,2	233,8	295,2		100	285,9	182,1	250,3	321,6	
Lombardia	80	220,8	134,6	191,3	250,3		81	266,1	176,0	227,8	304,4	
Piemonte	93	254,2	175,4	218,5	289,8		97	256,5	146,4	227,4	285,6	
Sicilia	93	268,4	162,2	235,4	301,4		80	220,4	134,1	191,0	249,8	
Toscana	98,0	247,8	163,2	215,5	280,1		82	307,1	178,8	268,4	345,8	
	**Vegetable and Fruit (g/day)**											
	N	Mean	SD	CI 95%		anova p-value	N	Mean	SD	CI 95%		anova p-value
Italian Region												
Basilicata	88	434,0	214,8	389,1	478,9	0,0026	95	466,4	208,4	424,5	508,4	0,0026
Calabria	75	499,4	280,3	436,0	562,9		67	449,0	215,3	397,5	500,6	
Emilia-Romagna	86	448,2	253,7	394,6	501,8		81	484,5	203,0	440,3	528,7	
Lazio	82	465,4	209,5	420,1	510,7		102	478,4	215,9	436,5	520,3	
Liguria	98	434,2	200,9	394,5	474,0		100	461,8	244,0	414,0	509,6	
Lombardia	80	364,3	188,4	323,0	405,5		81	433,5	213,7	387,0	480,0	
Piemonte	93	437,2	218,2	392,9	481,6		97	460,1	186,7	422,9	497,3	
Sicilia	93	420,1	193,9	380,7	459,5		80	377,8	173,1	339,9	415,8	
Toscana	98	430,6	210,2	388,9	472,2		82	517,5	236,5	466,4	568,7	
	**Fish (g/day)**											
	N	Mean	SD	CI 95%		anova p-value	N	Mean	SD	CI 95%		anova p-value
Italian Region												
Basilicata	88	46,1	38,3	38,1	54,1	<0,0001	95	43,4	31,3	37,1	49,7	<0,0001
Calabria	75	48,8	35,5	40,8	56,8		67	44,9	37,7	35,9	53,9	
Emilia-Romagna	86	51,9	31,0	45,4	58,4		81	45,3	32,6	38,2	52,4	
Lazio	82	50,7	35,0	43,1	58,2		102	42,6	26,2	37,5	47,7	
Liguria	98	56,1	41,8	47,9	64,4		100	48,4	31,0	42,3	54,5	
Lombardia	80	36,4	25,0	31,0	41,9		81	40,7	26,4	35,0	46,5	
Piemonte	93	44,6	41,2	36,3	53,0		97	36,2	25,5	31,1	41,3	
Sicilia	93	41,9	33,2	35,2	48,7		80	38,8	23,6	33,7	44,0	
Toscana	98	33,5	19,7	29,5	37,4		82	43,8	39,7	35,2	52,4	
	**Cheese (g/day)**											
	N	Mean	SD	CI 95%		anova p-value	N	Mean	SD	CI 95%		anova p-value
Italian Region												
Basilicata	88	43,2	36,3	35,6	50,7	0,0445	95	33,6	26,8	28,2	39,0	0,0445
Calabria	75	35,1	30,2	28,3	42,0		67	41,7	32,7	33,8	49,5	
Emilia-Romagna	86	54,3	43,9	45,0	63,6		81	41,5	39,4	32,9	50,1	
Lazio	82	41,1	34,4	33,6	48,5		102	35,4	28,2	29,9	40,8	
Liguria	98	46,8	36,0	39,7	53,9		100	36,7	35,6	29,7	43,7	
Lombardia	80	43,8	34,8	36,2	51,4		81	38,1	28,0	32,0	44,2	
Piemonte	93	40,5	29,6	34,5	46,6		97	37,9	29,0	32,1	43,7	
Sicilia	93	42,8	31,5	36,4	49,2		80	30,3	29,5	23,8	36,7	
Toscana	98	36,3	26,4	31,1	41,6		82	39,2	30,3	32,6	45,8	

For vegetables intake, the following foods were considered: leafy vegetables-cooked, leafy vegetables-raw, other vegetables, tomatoes-raw, tomatoes-cooked, root vegetables, cabbages, mushrooms, grain and pod vegetables, onion, garlic, stalk vegetables, mixed salad, mixed vegetables.
For vegetables intake were considered the following foods: leafy vegetable-cooked, leafy vegetable-raw, other vegetables, tomatoes-raw, tomatoes-cooked, root vegetables, cabbages, mushrooms, grain and pod vegetables, onion, garlic, stalk vegetables, mixed salad, mixed vegetables.
For fruits intake, the following foods were considered: citrus fruits and other fruits.
For fish intake, the following foods were considered: fish, crustaceans and molluscs.
For cheese intake, the following foods were considered: cheeses (including fresh cheeses).
Dietary information was collected by the self-administered Italian version of the food frequency questionnaire (FFQ) of the European Prospective Investigation into Cancer (EPIC).
SD: standard deviation; CI: confidence interval.
Means and standard deviations were age standardized by Italian National Institute of Statistics-ISTAT Italian population 2019 (except when they are reported by age-classes).
Anova to compare mean values among classes.

Table S.7. Age-standardized (Italian population) food group intake (EPIC questionnaire): processed meat, sweets/cakes, sweet drinks and alcohol mean by region.
Men and women residing in Italy aged 35-74 years, Health Examination Survey 2018-2019 - CUORE Project.

	Men						Women					
	**Processed meat (g/day)**											
	N	Mean	SD	CI 95%		anova p-value	N	Mean	SD	CI 95%		anova p-value
Italian Region												
Basilicata	88	42,4	37,4	34,6	50,2	0,0003	95	26,1	21,5	21,7	30,4	0,0003
Calabria	75	40,3	31,5	33,2	47,5		67	26,5	22,1	21,2	31,8	
Emilia-Romagna	86	42,1	31,4	35,5	48,8		81	27,3	22,2	22,5	32,1	
Lazio	82	36,2	26,8	30,4	42,0		102	26,5	22,2	22,2	30,8	
Liguria	98	30,0	24,0	25,3	34,8		100	22,7	19,3	19,0	26,5	
Lombardia	80	31,4	21,0	26,8	36,0		81	24,8	20,2	20,4	29,2	
Piemonte	93	30,5	23,7	25,7	35,3		97	20,9	23,4	16,3	25,6	
Sicilia	93	41,9	30,4	35,7	48,1		80	25,1	22,8	20,1	30,1	
Toscana	98	33,4	27,1	28,1	38,8		82	20,6	20,6	16,1	25,0	
	**Sweets/Cakes (g/day)**											
	N	Mean	SD	CI 95%		anova p-value	N	Mean	SD	CI 95%		anova p-value
Italian Region												
Basilicata	88	83,4	55,4	71,8	95,0	0,0630	95	83,2	66,4	69,8	96,5	0,0630
Calabria	75	79,5	67,7	64,2	94,9		67	73,2	74,3	55,4	91,0	
Emilia-Romagna	86	93,9	62,5	80,7	107,1		81	89,0	66,9	74,5	103,6	
Lazio	82	75,5	51,0	64,5	86,6		102	64,5	44,7	55,9	73,2	
Liguria	98	91,0	66,3	77,8	104,1		100	73,0	57,5	61,8	84,3	
Lombardia	80	87,5	61,9	73,9	101,1		81	85,6	47,0	75,4	95,9	
Piemonte	93	78,6	51,2	68,2	89,0		97	76,8	63,0	64,3	89,4	
Sicilia	93	85,0	63,7	72,1	98,0		80	69,5	60,6	56,2	82,8	
Toscana	98	81,4	56,4	70,3	92,6		82	84,9	72,3	69,3	100,6	
	**Sweet Drinks (ml/day)**											
	N	Mean	SD	CI 95%		anova p-value	N	Mean	SD	CI 95%		anova p-value
Italian Region												
Basilicata	88	119,3	161,4	85,6	153,0	0,4315	95	89,8	172,6	55,1	124,5	0,4315
Calabria	75	124,7	209,9	77,2	172,2		67	57,7	83,7	37,6	77,7	
Emilia-Romagna	86	93,6	140,2	64,0	123,3		81	89,8	146,4	58,0	121,7	
Lazio	82	104,2	178,4	65,6	142,8		102	80,6	138,8	53,6	107,5	
Liguria	98	104,7	161,9	72,7	136,8		100	68,9	95,8	50,1	87,7	
Lombardia	80	96,3	140,4	65,5	127,0		81	65,5	112,9	40,9	90,1	
Piemonte	93	79,4	110,0	57,1	101,8		97	72,3	155,0	41,4	103,1	
Sicilia	93	116,6	216,6	72,6	160,6		80	58,8	107,8	35,2	82,4	
Toscana	98	66,3	80,5	50,4	82,3		82	63,6	117,0	38,2	88,9	
	**Alcohol (g/day)**											
	N	Mean	SD	CI 95%		anova p-value	N	Mean	SD	CI 95%		anova p-value
Italian Region												
Basilicata	88	18,6	22,3	13,9	23,2	0,0007	95	4,5	8,2	2,9	6,2	0,0007
Calabria	75	14,5	16,4	10,8	18,2		67	2,5	6,5	1,0	4,1	
Emilia-Romagna	86	17,5	14,9	14,4	20,7		81	5,6	11,2	3,2	8,1	
Lazio	82	9,2	11,7	6,7	11,7		102	5,6	8,5	4,0	7,3	
Liguria	98	15,1	16,1	11,9	18,3		100	6,8	10,7	4,7	8,9	
Lombardia	80	16,6	18,9	12,5	20,8		81	5,6	9,0	3,6	7,5	
Piemonte	93	15,8	14,8	12,7	18,8		97	8,5	10,4	6,4	10,6	
Sicilia	93	10,3	13,1	7,7	13,0		80	3,1	6,4	1,8	4,5	
Toscana	98	14,5	15,4	11,5	17,5		82	6,1	8,0	4,3	7,8	

For processed meat intake, the following foods were considered: sausages, salami and other preserved meat.
For sweets/cakes intake, the following foods were considered: sugar, honey, jam, chocolate, candy bars, paste, confetti/flakes, confectionery non chocolate, ice cream, cakes, pies, pastries, puddings (not milk based), dry cakes, biscuits.
For sweet beverages intake, the following foods were considered: fruit and vegetable juices, carbonated/soft/isotonic drinks, diluted syrups.
For alcohol intake, the following foods were considered: alcoholic beverages.
Dietary information was collected by the self-administered Italian version of the food frequency questionnaire (FFQ) of the European Prospective Investigation into Cancer (EPIC).
SD: standard deviation; CI: confidence interval.
Means and standard deviations were age standardized by Italian National Institute of Statistics-ISTAT Italian population 2019 (except when they are reported by age-classes).
Anova to compare mean values among classes.

Table S.8. Age-standardized (Italian population) food group intake (EPIC questionnaire): cereals, potatoes, legumes and oil mean by region.
Men and women residing in Italy aged 35-74 years, Health Examination Survey 2018-2019 - CUORE Project.

	Men						Women					
**Cereals (g/day)**												
	N	Mean	SD	CI 95%		anova p-value	N	Mean	SD	CI 95%		anova p-value
Italian Region												
Basilicata	88	178,8	96,2	158,7	198,9	0,0202	95	148,1	85,2	131,0	165,3	0,0202
Calabria	75	153,6	89,1	133,4	173,7		67	135,5	84,4	115,3	155,7	
Emilia-Romagna	86	206,1	97,4	185,5	226,7		81	135,1	87,9	116,0	154,2	
Lazio	82	161,4	83,6	143,3	179,4		102	138,3	82,8	122,2	154,3	
Liguria	98	182,2	96,9	163,0	201,4		100	124,6	80,5	108,8	140,4	
Lombardia	80	171,5	90,9	151,6	191,4		81	124,5	70,0	109,2	139,7	
Piemonte	93	165,9	109,0	143,7	188,0		97	143,1	98,6	123,5	162,8	
Sicilia	93	179,4	95,9	159,9	198,9		80	118,0	86,8	99,0	137,1	
Toscana	98	189,2	90,4	171,3	207,1		82	152,9	81,1	135,4	170,5	
**Potatoes (g/day)**												
	N	Mean	SD	CI 95%		anova p-value	N	Mean	SD	CI 95%		anova p-value
Italian Region												
Basilicata	88	22,2	17,6	18,6	25,9	0,0077	95	22,8	18,6	19,1	26,6	0,0077
Calabria	75	26,9	23,5	21,6	32,3		67	30,7	42,8	20,5	41,0	
Emilia-Romagna	86	22,8	21,1	18,3	27,3		81	19,9	19,1	15,8	24,1	
Lazio	82	25,6	22,4	20,8	30,4		102	18,1	17,8	14,6	21,5	
Liguria	98	23,6	21,6	19,4	27,9		100	20,7	20,9	16,6	24,8	
Lombardia	80	21,3	18,9	17,2	25,5		81	17,2	14,2	14,1	20,2	
Piemonte	93	21,6	19,1	17,8	25,5		97	19,4	18,7	15,7	23,2	
Sicilia	93	21,2	16,1	17,9	24,5		80	21,0	19,7	16,7	25,3	
Toscana	98	21,6	18,5	17,9	25,2		82	21,5	22,3	16,7	26,3	
**Legumes (g/day)**												
	N	Mean	SD	CI 95%		anova p-value	N	Mean	SD	CI 95%		anova p-value
Italian Region												
Basilicata	88	28,7	17,2	25,1	32,3	<0,0001	95	28,0	13,8	25,3	30,8	<0,0001
Calabria	75	31,4	26,9	25,3	37,5		67	29,9	21,4	24,8	35,0	
Emilia-Romagna	86	17,6	15,5	14,3	20,9		81	18,6	17,3	14,9	22,4	
Lazio	82	23,7	22,2	18,9	28,5		102	21,2	17,5	17,8	24,6	
Liguria	98	19,7	19,5	15,8	23,5		100	13,3	12,9	10,8	15,8	
Lombardia	80	15,4	14,9	12,1	18,6		81	17,4	13,3	14,5	20,3	
Piemonte	93	18,5	18,0	14,8	22,2		97	16,3	12,2	13,9	18,8	
Sicilia	93	18,8	13,0	16,2	21,5		80	16,2	11,1	13,7	18,6	
Toscana	98	22,1	16,1	18,9	25,2		82	17,6	15,0	14,3	20,8	
**Oil (g/day)**												
	N	Mean	SD	CI 95%		anova p-value	N	Mean	SD	CI 95%		anova p-value
Italian Region												
Basilicata	88	15,6	10,9	13,3	17,8	<0,0001	95	17,6	9,3	15,7	19,5	<0,0001
Calabria	75	13,5	12,3	10,7	16,3		67	14,8	10,7	12,2	17,3	
Emilia-Romagna	86	16,1	12,2	13,5	18,7		81	16,5	12,2	13,8	19,1	
Lazio	82	18,8	11,4	16,4	21,3		102	19,5	10,5	17,5	21,6	
Liguria	98	16,7	11,8	14,3	19,0		100	17,0	12,2	14,6	19,4	
Lombardia	80	14,0	10,3	11,8	16,3		81	16,1	11,6	13,6	18,6	
Piemonte	93	18,4	11,1	16,2	20,7		97	19,6	10,1	17,6	21,6	
Sicilia	93	11,6	10,1	9,6	13,7		80	12,5	10,4	10,2	14,7	
Toscana	98	17,7	10,8	15,6	19,9		82	21,5	11,9	18,9	24,1	

For cereals intake, the following foods were considered: pasta, rice, white and whole meal bread, other grains, crispbread, rusks, and breakfast cereals.
For potatoes intake, the following foods were considered: french fries, boiled potatoes, roasted potatoes, pure potatoes, croquette potatoes.
Dietary information was collected by the self-administered Italian version of the food frequency questionnaire (FFQ) of the European Prospective Investigation into Cancer (EPIC).
SD: standard deviation; CI: confidence interval.
Means and standard deviations were age standardized by Italian National Institute of Statistics-ISTAT Italian population 2019 (except when they are reported by age-classes).
Anova to compare mean values among classes.

Table S.9. Age-standardized (Italian population) food group intake (EPIC questionnaire): meat, eggs, milk mean by region.
Men and women residing in Italy aged 35-74 years, Health Examination Survey 2018-2019 - CUORE Project.

	Men						Women					
	**Meat (g/day)**											
	N	Mean	SD	CI 95%		anova p-value	N	Mean	SD	CI 95%		anova p-value
Italian Region												
Basilicata	88	87,6	52,5	76,6	98,6	0,0132	95	78,4	41,7	70,0	86,8	0,0132
Calabria	75	87,9	68,6	72,4	103,4		67	77,4	50,5	65,3	89,5	
Emilia-Romagna	86	111,1	61,7	98,1	124,2		81	90,8	53,9	79,1	102,5	
Lazio	82	94,1	51,2	83,0	105,2		102	84,7	49,3	75,2	94,3	
Liguria	98	83,5	49,4	73,8	93,3		100	77,6	52,5	67,3	87,8	
Lombardia	80	99,0	61,7	85,5	112,5		81	74,3	45,7	64,3	84,2	
Piemonte	93	84,2	54,0	73,2	95,2		97	80,9	56,0	69,7	92,0	
Sicilia	93	109,4	56,4	97,9	120,9		80	76,7	51,9	65,3	88,1	
Toscana	98	94,5	58,9	82,9	106,2		82	82,6	48,3	72,1	93,1	
	**Eggs (g/day)**											
	N	Mean	SD	CI 95%		anova p-value	N	Mean	SD	CI 95%		anova p-value
Italian Region												
Basilicata	88	11,6	11,2	9,3	14,0	0,2727	95	8,5	6,2	7,2	9,7	0,2727
Calabria	75	10,2	9,5	8,0	12,3		67	9,3	7,4	7,5	11,1	
Emilia-Romagna	86	10,8	13,8	7,9	13,7		81	10,9	11,1	8,5	13,3	
Lazio	82	11,3	9,1	9,3	13,3		102	10,6	8,3	9,0	12,3	
Liguria	98	10,4	8,6	8,7	12,1		100	10,1	9,1	8,3	11,9	
Lombardia	80	9,3	8,1	7,5	11,1		81	9,4	7,1	7,8	10,9	
Piemonte	93	11,1	9,8	9,1	13,1		97	12,2	10,5	10,1	14,3	
Sicilia	93	12,8	9,4	10,9	14,7		80	10,0	7,3	8,4	11,6	
Toscana	98	10,4	10,0	8,4	12,4		82	9,3	7,7	7,7	11,0	
	**Milk (ml/day)**											
	N	Mean	SD	CI 95%		anova p-value	N	Mean	SD	CI 95%		anova p-value
Italian Region												
Basilicata	88	71,2	97,6	50,8	91,6	0,6302	95	114,0	112,6	91,4	136,7	0,6302
Calabria	75	79,4	139,1	48,0	110,9		67	108,7	128,8	77,9	139,5	
Emilia-Romagna	86	73,7	97,7	53,0	94,3		81	90,9	108,1	67,4	114,5	
Lazio	82	79,6	93,8	59,3	99,9		102	93,7	104,3	73,5	114,0	
Liguria	98	93,0	119,4	69,3	116,6		100	92,0	115,8	69,3	114,7	
Lombardia	80	80,6	88,8	61,2	100,1		81	110,1	133,2	81,1	139,1	
Piemonte	93	78,7	128,0	52,7	104,7		97	80,2	94,2	61,4	98,9	
Sicilia	93	88,5	112,1	65,7	111,3		80	87,4	129,9	59,0	115,9	
Toscana	98	80,9	109,6	59,2	102,6		82	126,1	129,2	98,1	154,0	

For meats intake, the following foods were considered: beef, veal, pork, horse, chicken, turkey, rabbit (domestic).
Dietary information was collected by the self-administered Italian version of the food frequency questionnaire (FFQ) of the European Prospective Investigation into Cancer (EPIC).
SD: standard deviation; CI: confidence interval.
Means and standard deviations were age standardized by Italian National Institute of Statistics-ISTAT Italian population 2019 (except when they are reported by age-classes).
Anova to compare mean values among classes.

Table S.10. Age-standardized (Italian population) nutrients intake (EPIC questionnaire): proteins (total, animal and vegetable) mean by region.
Men and women residing in Italy aged 35-74 years, Health Examination Survey 2018-2019 - CUORE Project.

	Men						Women					
	**Proteins Tot (% total Kcal)**											
	N	Mean	SD	CI 95%		anova p-value	N	Mean	SD	CI 95%		anova p-value
Italian Region												
Basilicata	88	14,9	2,3	14,5	15,4	0,001	95	16,0	4,3	15,2	16,9	0,001
Calabria	75	16,2	6,0	14,8	17,5		67	16,4	3,4	15,6	17,2	
Emilia-Romagna	86	15,6	2,5	15,0	16,1		81	16,2	3,2	15,5	16,9	
Lazio	82	16,0	2,4	15,5	16,5		102	16,6	4,6	15,7	17,5	
Liguria	98	15,1	2,3	14,6	15,6		100	16,0	3,5	15,3	16,7	
Lombardia	80	15,4	2,9	14,8	16,1		81	15,6	3,1	14,9	16,2	
Piemonte	93	15,2	2,8	14,6	15,7		97	15,8	3,2	15,2	16,5	
Sicilia	93	16,2	2,7	15,7	16,7		80	16,6	3,4	15,9	17,3	
Toscana	98	15,0	2,5	14,5	15,5		82	15,2	2,6	14,7	15,8	
	**Animal proteins (% total Kcal)**											
	N	Mean	SD	CI 95%		anova p-value	N	Mean	SD	CI 95%		anova p-value
Italian Region												
Basilicata	88	9,8	3,0	9,2	10,4	0,0032	95	10,6	4,6	9,6	11,5	0,0032
Calabria	75	11,1	6,6	9,7	12,6		67	11,1	4,3	10,1	12,1	
Emilia-Romagna	86	10,5	3,2	9,9	11,2		81	11,2	3,8	10,4	12,0	
Lazio	82	10,8	2,8	10,2	11,4		102	11,3	5,3	10,3	12,3	
Liguria	98	10,1	2,9	9,5	10,6		100	11,1	4,1	10,3	11,9	
Lombardia	80	10,5	3,2	9,8	11,2		81	10,6	3,3	9,8	11,3	
Piemonte	93	10,1	3,6	9,3	10,8		97	10,4	3,9	9,6	11,1	
Sicilia	93	11,0	3,3	10,4	11,7		80	11,4	3,8	10,6	12,2	
Toscana	98	9,6	3,2	8,9	10,2		82	9,9	3,2	9,2	10,6	
	**Vegetable proteins (% total Kcal)**											
	N	Mean	SD	CI 95%		anova p-value	N	Mean	SD	CI 95%		anova p-value
Italian Region												
Basilicata	88	5,1	1,4	4,8	5,4	0,0015	95	5,5	1,3	5,2	5,7	0,0015
Calabria	75	5,0	1,1	4,8	5,3		67	5,3	1,5	5,0	5,7	
Emilia-Romagna	86	5,0	1,1	4,8	5,3		81	5,0	1,2	4,7	5,2	
Lazio	82	5,2	1,0	5,0	5,4		102	5,3	1,2	5,1	5,6	
Liguria	98	5,0	1,1	4,8	5,3		100	4,9	1,1	4,7	5,1	
Lombardia	80	5,0	1,0	4,7	5,2		81	5,0	1,0	4,8	5,2	
Piemonte	93	5,1	1,3	4,9	5,4		97	5,5	1,3	5,2	5,7	
Sicilia	93	5,1	1,3	4,9	5,4		80	5,2	1,3	4,9	5,5	
Toscana	98	5,5	1,2	5,2	5,7		82	5,3	1,1	5,0	5,5	

Dietary information was collected by the self-administered Italian version of the food frequency questionnaire (FFQ) of the European Prospective Investigation into Cancer (EPIC).
SD: standard deviation; CI: confidence interval.
Means and standard deviations were age standardized by Italian National Institute of Statistics-ISTAT Italian population 2019 (except when they are reported by age-classes).
Anova to compare mean values among classes.

Table S.11. Age-standardized (Italian population) nutrients intake (EPIC questionnaire): lipids (animal and vegetable) and cholesterol mean by region.
Men and women residing in Italy aged 35-74 years, Health Examination Survey 2018-2019 - CUORE Project.

	Men						Women					
**Animal Lipids (% total Kcal)**												
	N	Mean	SD	CI 95%		anova p-value	N	Mean	SD	CI 95%		anova p-value
Italian Region												
Basilicata	88	18,0	6,0	16,7	19,2	0,0017	95	17,0	4,5	16,1	17,9	0,0017
Calabria	75	17,9	5,1	16,8	19,1		67	18,6	6,7	17,0	20,3	
Emilia-Romagna	86	18,5	5,2	17,4	19,6		81	18,6	4,6	17,6	19,5	
Lazio	82	17,8	5,1	16,7	18,9		102	16,9	5,2	15,9	17,9	
Liguria	98	18,3	5,0	17,3	19,3		100	17,7	5,1	16,6	18,7	
Lombardia	80	17,9	4,7	16,9	19,0		81	18,1	4,6	17,1	19,1	
Piemonte	93	17,3	5,3	16,2	18,4		97	16,8	4,9	15,8	17,8	
Sicilia	93	19,1	5,6	18,0	20,2		80	18,4	5,5	17,2	19,6	
Toscana	98	16,7	5,4	15,6	17,8		82	16,8	5,0	15,8	17,9	
**Vegetable Lipids (% total Kcal)**												
	N	Mean	SD	CI 95%		anova p-value	N	Mean	SD	CI 95%		anova p-value
Italian Region												
Basilicata	88	15,9	5,6	14,7	17,0	0,0009	95	18,9	4,8	17,9	19,9	0,0009
Calabria	75	16,9	5,2	15,7	18,0		67	19,4	6,1	18,0	20,9	
Emilia-Romagna	86	16,5	4,9	15,5	17,6		81	18,5	5,8	17,3	19,8	
Lazio	82	17,9	5,4	16,8	19,1		102	19,9	6,3	18,7	21,2	
Liguria	98	17,3	4,9	16,3	18,3		100	20,9	7,3	19,5	22,4	
Lombardia	80	16,1	7,6	14,4	17,7		81	20,0	6,1	18,7	21,3	
Piemonte	93	18,7	5,7	17,5	19,8		97	20,8	6,9	19,5	22,2	
Sicilia	93	16,6	5,3	15,5	17,6		80	20,1	6,6	18,6	21,5	
Toscana	98	18,3	6,4	17,0	19,5		82	20,5	6,7	19,1	22,0	
**Cholesterol (mg/day)**												
	N	Mean	SD	CI 95%		anova p-value	N	Mean	SD	CI 95%		anova p-value
Italian Region												
Basilicata	88	151,6	42,4	142,7	160,4	0,0023	95	157,0	42,6	148,4	165,5	0,0023
Calabria	75	164,7	71,4	148,6	180,9		67	164,8	63,9	149,5	180,1	
Emilia-Romagna	86	150,7	44,6	141,3	160,1		81	168,8	43,3	159,4	178,2	
Lazio	82	155,0	41,0	146,2	163,9		102	155,7	51,0	145,7	165,6	
Liguria	98	152,8	40,3	144,8	160,8		100	164,7	49,9	154,9	174,5	
Lombardia	80	148,5	40,9	139,6	157,5		81	159,8	62,3	146,3	173,4	
Piemonte	93	149,9	46,5	140,4	159,3		97	160,5	51,8	150,2	170,8	
Sicilia	93	169,1	46,5	159,6	178,5		80	174,2	63,5	160,3	188,1	
Toscana	98	148,0	52,3	137,6	158,4		82	150,4	48,1	140,0	160,8	

Dietary information was collected by the self-administered Italian version of the food frequency questionnaire (FFQ) of the European Prospective Investigation into Cancer (EPIC).
SD: standard deviation; CI: confidence interval.
Means and standard deviations were age standardized by Italian National Institute of Statistics-ISTAT Italian population 2019 (except when they are reported by age-classes).
Anova to compare mean values among classes.

Table S.12. Age-standardized (Italian population) nutrients intake (EPIC questionnaire): saturated, polyunsaturated and monounsaturated fat, lipids total mean by region.
Men and women residing in Italy aged 35-74 years, Health Examination Survey 2018-2019 - CUORE Project.

	Men						Women					
**Saturated fat (% total Kcal)**												
	N	Mean	SD	CI 95%		anova p-value	N	Mean	SD	CI 95%		anova p-value
Italian Region												
Basilicata	88	10,7	2,9	10,1	11,3	0,0064	95	10,9	2,2	10,4	11,3	0,0064
Calabria	75	10,7	2,0	10,2	11,1		67	12,0	3,1	11,2	12,7	
Emilia-Romagna	86	11,3	2,4	10,8	11,8		81	11,7	2,4	11,1	12,2	
Lazio	82	10,8	2,5	10,2	11,3		102	10,8	2,2	10,4	11,3	
Liguria	98	11,3	2,3	10,8	11,7		100	11,5	2,3	11,0	11,9	
Lombardia	80	10,9	2,3	10,5	11,4		81	11,8	2,1	11,3	12,3	
Piemonte	93	11,1	2,5	10,5	11,6		97	11,2	2,2	10,8	11,6	
Sicilia	93	11,2	2,5	10,7	11,7		80	11,7	2,9	11,1	12,4	
Toscana	98	10,5	2,5	10,1	11,0		82	11,2	2,4	10,7	11,7	
**Polyunsaturated fat (% total Kcal)**												
	N	Mean	SD	CI 95%		anova p-value	N	Mean	SD	CI 95%		anova p-value
Italian Region												
Basilicata	88	4,8	1,7	4,4	5,1	0,2010	95	5,1	1,2	4,9	5,4	0,2010
Calabria	75	5,3	1,3	5,0	5,6		67	5,3	1,2	5,0	5,6	
Emilia-Romagna	86	5,0	1,1	4,8	5,3		81	5,3	1,2	5,1	5,6	
Lazio	82	5,2	1,3	5,0	5,5		102	5,2	1,2	5,0	5,5	
Liguria	98	4,8	1,2	4,6	5,0		100	5,4	1,4	5,1	5,7	
Lombardia	80	4,9	1,5	4,6	5,2		81	5,6	1,8	5,2	6,0	
Piemonte	93	5,0	1,3	4,7	5,3		97	5,4	1,5	5,1	5,7	
Sicilia	93	5,2	1,4	4,9	5,4		80	5,5	1,4	5,2	5,8	
Toscana	98	5,0	1,2	4,8	5,3		82	5,2	1,3	4,9	5,4	
**Monounsaturated fat (% total Kcal)**												
	N	Mean	SD	CI 95%		anova p-value	N	Mean	SD	CI 95%		anova p-value
Italian Region												
Basilicata	88	15,5	4,0	14,7	16,3	0,0134	95	17,0	3,2	16,3	17,6	0,0134
Calabria	75	16,0	3,4	15,3	16,8		67	17,8	4,1	16,8	18,7	
Emilia-Romagna	86	15,7	2,8	15,1	16,3		81	16,9	4,1	16,0	17,8	
Lazio	82	16,6	3,3	15,9	17,3		102	17,6	3,5	16,9	18,2	
Liguria	98	16,5	3,3	15,8	17,1		100	18,4	4,9	17,5	19,4	
Lombardia	80	15,3	4,6	14,3	16,3		81	17,5	3,5	16,7	18,2	
Piemonte	93	16,8	3,6	16,1	17,6		97	17,8	4,1	17,0	18,6	
Sicilia	93	16,3	3,8	15,5	17,1		80	18,0	4,6	17,0	19,0	
Toscana	98	16,5	3,9	15,7	17,3		82	17,9	4,2	17,0	18,8	
**Lipids Tot (% total Kcal)**												
	N	Mean	SD	CI 95%		anova p-value	N	Mean	SD	CI 95%		anova p-value
Italian Region												
Basilicata	88	33,8	8,1	32,1	35,5	0,0534	95	35,9	5,9	34,7	37,1	0,0534
Calabria	75	34,8	6,1	33,4	36,2		67	38,1	7,0	36,4	39,8	
Emilia-Romagna	86	35,0	5,1	33,9	36,1		81	37,1	6,9	35,6	38,5	
Lazio	82	35,7	6,1	34,4	37,1		102	36,8	5,6	35,7	37,9	
Liguria	98	35,6	5,6	34,5	36,7		100	38,6	7,1	37,2	40,0	
Lombardia	80	34,0	7,3	32,4	35,6		81	38,1	5,3	36,9	39,3	
Piemonte	93	35,9	6,5	34,6	37,3		97	37,7	6,7	36,3	39,0	
Sicilia	93	35,7	7,1	34,2	37,1		80	38,5	7,7	36,8	40,1	
Toscana	98	35,0	6,8	33,6	36,3		82	37,4	6,4	36,0	38,7	

Dietary information was collected by the self-administered Italian version of the food frequency questionnaire (FFQ) of the European Prospective Investigation into Cancer (EPIC).
SD: standard deviation; CI: confidence interval.
Means and standard deviations were age standardized by Italian National Institute of Statistics-ISTAT Italian population 2019 (except when they are reported by age-classes).
Anova to compare mean values among classes.

Table S.13. Age-standardized (Italian population) nutrients intake (EPIC questionnaire) fiber, sodium, potassium, carbohydrates and simple carbohydrates mean by region.
Men and women residing in Italy aged 35-74 years, Health Examination Survey 2018-2019 - CUORE Project.

	Men						Women					
	**Fiber (g/day)**											
	N	Mean	SD	CI 95%		anova p-value	N	Mean	SD	CI 95%		anova p-value
Italian Region												
Basilicata	88	9,7	2,8	9,2	10,3	<0,0001	95,0	11,6	2,9	11,0	12,2	<0,0001
Calabria	75	10,4	2,9	9,8	11,1		67,0	11,4	2,8	10,7	12,1	
Emilia-Romagna	86	9,3	2,6	8,8	9,9		81,0	11,0	3,1	10,3	11,6	
Lazio	82	10,4	3,0	9,8	11,1		102,0	11,6	3,1	11,0	12,2	
Liguria	98	9,4	2,5	8,9	9,9		100,0	10,7	2,7	10,1	11,2	
Lombardia	80	9,1	2,2	8,6	9,6		81,0	10,9	3,0	10,2	11,6	
Piemonte	93	10,3	3,2	9,7	11,0		97,0	11,8	3,4	11,1	12,5	
Sicilia	93	9,5	2,3	9,1	10,0		80,0	10,9	2,6	10,3	11,5	
Toscana	98	10,3	2,7	9,8	10,9		82,0	11,5	2,8	10,9	12,1	
	**Sodium (g/day)**											
	N	Mean	SD	CI 95%		anova p-value	N	Mean	SD	CI 95%		anova p-value
Italian Region												
Basilicata	88	2,7	1,1	2,4	2,9	0,001	95	2,1	1,0	1,9	2,3	0,001
Calabria	75	2,4	1,1	2,1	2,6		67	2,1	0,9	1,9	2,3	
Emilia-Romagna	86	3,0	1,1	2,7	3,2		81	2,3	1,1	2,0	2,5	
Lazio	82	2,4	0,9	2,2	2,6		102	2,1	1,0	1,9	2,3	
Liguria	98	2,5	1,0	2,3	2,7		100	2,0	0,9	1,9	2,2	
Lombardia	80	2,4	0,9	2,2	2,6		81	2,1	0,7	1,9	2,2	
Piemonte	93	2,3	0,9	2,1	2,5		97	2,0	1,0	1,8	2,2	
Sicilia	93	2,6	1,1	2,3	2,8		80	1,9	1,0	1,6	2,1	
Toscana	98	2,5	0,9	2,3	2,6		82	2,2	0,9	2,0	2,4	
	**Potassium (g/day)**											
	N	Mean	SD	CI 95%		anova p-value	N	Mean	SD	CI 95%		anova p-value
Italian Region												
Basilicata	88	3,2	1,2	2,9	3,4	0,0100	95	3,1	1,1	2,9	3,3	0,0096
Calabria	75	3,4	1,4	3,0	3,7		67	3,0	1,0	2,8	3,3	
Emilia-Romagna	86	3,5	1,1	3,2	3,7		81	3,1	1,1	2,9	3,4	
Lazio	82	3,2	0,8	3,0	3,3		102	3,0	0,9	2,8	3,2	
Liguria	98	3,2	1,2	3,0	3,5		100	3,0	1,1	2,8	3,2	
Lombardia	80	2,9	0,8	2,7	3,1		81	2,9	0,9	2,7	3,1	
Piemonte	93	3,1	0,9	2,9	3,3		97	3,0	0,9	2,9	3,2	
Sicilia	93	3,1	1,0	2,9	3,3		80	2,6	1,0	2,4	2,9	
Toscana	98	3,1	0,9	3,0	3,3		82	3,2	1,1	3,0	3,5	
	**Carbohydrate (% total Kcal)**											
	N	Mean	SD	CI 95%		anova p-value	N	Mean	SD	CI 95%		anova p-value
Italian Region												
Basilicata	88	48,8	10,9	46,5	51,1	0,1206	95	49,6	8,5	47,9	51,3	0,1206
Calabria	75	47,9	9,9	45,6	50,1		67	47,7	9,4	45,5	50,0	
Emilia-Romagna	86	47,2	7,3	45,6	48,7		81	47,8	9,5	45,8	49,9	
Lazio	82	48,3	7,6	46,6	49,9		102	47,6	7,8	46,1	49,1	
Liguria	98	48,0	7,5	46,5	49,5		100	46,0	9,4	44,1	47,8	
Lombardia	80	48,4	9,4	46,4	50,5		81	47,4	7,4	45,8	49,1	
Piemonte	93	46,3	9,4	44,4	48,3		97	46,3	9,1	44,5	48,2	
Sicilia	93	48,2	9,8	46,2	50,2		80	46,5	9,9	44,4	48,7	
Toscana	98	48,4	8,7	46,7	50,2		82	48,0	8,5	46,2	49,8	
	**Simple carbohydrates (% total Kcal)**											
	N	Mean	SD	CI 95%		anova p-value	N	Mean	SD	CI 95%		anova p-value
Italian Region												
Basilicata	88	18,6	6,7	17,2	20,0	0,5132	95	20,5	6,2	19,3	21,7	0,5132
Calabria	75	19,5	8,1	17,7	21,4		67	19,0	6,9	17,3	20,6	
Emilia-Romagna	86	17,5	5,6	16,3	18,7		81	21,1	7,0	19,5	22,6	
Lazio	82	19,6	6,0	18,3	20,8		102	19,7	6,1	18,5	20,9	
Liguria	98	18,5	5,9	17,3	19,6		100	20,2	7,0	18,8	21,5	
Lombardia	80	19,1	6,3	17,8	20,5		81	21,4	7,1	19,9	23,0	
Piemonte	93	18,7	4,9	17,8	19,7		97	19,5	5,9	18,3	20,7	
Sicilia	93	18,4	7,0	17,0	19,8		80	19,3	7,0	17,8	20,9	
Toscana	98	17,6	5,1	16,6	18,6		82	20,4	6,7	19,0	21,9	

Dietary information was collected by the self-administered Italian version of the food frequency questionnaire (FFQ) of the European Prospective Investigation into Cancer (EPIC).
SD: standard deviation; CI: confidence interval.
Means and standard deviations were age standardized by Italian National Institute of Statistics-ISTAT Italian population 2019 (except when they are reported by age-classes).
Anova to compare mean values among classes.

Table S.14. Age-standardized (European standard population) current smokers and former smokers prevalence, and mean number of cigarettes smoked per day, by sex, age classes, geographical area and educational level. Cigarette, cigar and pipe smokers prevalence.
Men and women residing in Italy aged 35-74 years, Health Examination Survey 2018-2019 - CUORE Project.

	Men						Women					
**Current Smokers**												
	N	n	%	CI 95%		χ^2 p-value	N	n	%	CI 95%		χ^2 p-value
**All**	**1031**	**234**	**23,2**	**18,0**	**28,3**		**1059**	**196**	**18,68**	**13,98**	**23,39**	
**Age classes (years)**												
35-44	249	72	28,9	23,3	34,6	0,007	235	49	20,9	15,7	26,1	0,2763
45-54	248	63	25,4	20,0	30,8		271	48	17,7	13,2	22,3	
55-64	276	54	19,6	14,9	24,3		276	57	20,7	15,9	25,4	
65-74	258	45	17,4	12,8	22,1		277	42	15,2	10,9	19,4	
**Italian Area**												
North	409	88	21,7	13,7	29,8	0,1115	421	74	17,6	10,3	25,0	0,8172
Center	206	58	29,1	16,7	41,5		208	40	19,5	8,6	30,3	
South	416	88	21,6	13,7	29,5		430	82	19,3	11,9	26,8	
**Education**												
Primary and middle school	303	72	25,0	15,0	35,0	0,6559	338	65	19,7	11,0	28,4	0,7492
High school and university	728	162	22,4	16,4	28,5		720	131	18,3	12,7	23,9	
**Daily smoke - number of cigarettes**												
	N	Mean	SD	CI 95%		anova p-value	N	Mean	SD	CI 95%		anova p-value
**All**	**234**	**13,29**	**8,85**	**12,75**	**13,83**		**196**	**10,66**	**6,37**	**10,28**	**11,04**	
**Age classes (years)**												
35-44	72	12,3	8,4	11,3	13,4	0,0155	49	8,2	5,7	7,5	8,9	0,0155
45-54	63	14,1	9,9	12,9	15,3		48	12,0	7,3	11,1	12,8	
55-64	54	11,6	6,6	10,8	12,4		57	11,2	5,8	10,5	11,9	
65-74	45	16,3	10,2	15,0	17,5		42	11,4	5,9	10,7	12,1	
**Italian Area**												
North	88	12,8	9,8	11,8	13,7	0,5733	74	10,6	7,8	9,9	11,4	0,5733
Center	58	12,3	7,5	11,2	13,3		40	10,6	5,6	9,8	11,4	
South	88	14,6	8,6	13,7	15,4		82	10,7	5,4	10,2	11,3	
**Education**												
Primary and middle school	72	16,6	10,0	15,5	17,7	<0,0001	65	12,8	7,0	12,0	13,5	<0,0001
High school and university	162	11,9	7,9	11,3	12,4		131	9,6	5,8	9,2	10,1	
**Former Smokers**												
	N	n	%	CI 95%		χ^2 p-value	N	n	%	CI 95%		χ^2 p-value
**All**	**1031**	**351**	**43,0**	**36,9**	**49,0**		**1059**	**246**	**27,8**	**22,4**	**33,2**	
**Age classes (years)**												
35-44	249	59	23,7	18,4	29,0	<0.0001	235	44	18,7	13,7	23,7	<0.0001
45-54	248	68	27,4	21,9	33,0		271	42	15,5	11,2	19,8	
55-64	276	102	37,0	31,3	42,7		276	77	27,9	22,6	33,2	
65-74	258	122	47,3	41,2	53,4		277	83	30,0	24,6	35,4	
**Italian Area**												
North	409	130	39,9	30,3	49,4	0,0851	421	105	29,7	21,0	38,5	0,0148
Center	206	61	40,8	27,3	54,2		208	59	34,1	21,1	47,1	
South	416	160	47,0	37,4	56,6		430	82	22,8	14,9	30,8	
**Education**												
Primary and middle school	303	127	54,0	42,5	65,5	0,0001	338	65	23,3	14,1	32,6	0,0458
High school and university	728	224	38,7	31,7	45,8		720	181	29,7	23,1	36,3	
**Cigar Smokers**												
	N	n	%	CI 95%		χ^2 p-value	N	n	%	CI 95%		χ^2 p-value
**All**	**1031**	**115**	**13,1**	**9,0**	**17,2**	**<0,0001**	**1059**	**10**	**1,13**	**0**	**2,41**	**<0,0001**
**Pipe Smokers**												
	N	n	%	CI 95%		χ^2 p-value	N	n	%	CI 95%		χ^2 p-value
**All**	**1031**	**68**	**7,5**	**4,3**	**10,7**	**<0,0001**	**1059**	**7**	**0,8**	**0,0**	**1,8**	**<0,0001**
**Cigarette and Cigar Smokers**												
	N	n	%	CI 95%		χ^2 p-value	N	n	%	CI 95%		χ^2 p-value
**All**	**1031**	**38**	**3,7**	**1,4**	**6**	**<0,0001**	**1059**	**2**	**0,2**	**0**	**0,7**	**<0,0001**
**Cigarette and Pipe Smokers**												
	N	n	%	CI 95%		χ^2 p-value	N	n	%	CI 95%		χ^2 p-value
**All**	**1031**	**24**	**2,3**	**0,5**	**4,1**	**<0,0001**	**1059**	**3**	**0,3**	**0**	**0,9**	**<0,0001**

N: number of participants denominator.
n: number of participants related to the prevalence numerator.
Current smoker is defined as a person who smokes one or more cigarettes per day.
A former smoker is defined as a person who has quit smoking for at least 12 months.
Number of cigarettes refers to packaged, handmade or electronic cigarettes.
SD: standard deviation; CI: confidence interval.
Means, standard deviations, and prevalence were age standardized by European standard population distribution 2013 (except when they are reported by age-classes).
Anova to compare mean values among classes; chi-square test to compare prevalence among classes.
Pool of the following Italian regions: Piedmont, Lombardy, Liguria, Emilia Romagna, Tuscany, Lazio, Abruzzo, Basilicata, Calabria, and Sicily.

Italian Area: North (Piedmont, Lombardy, Liguria, Emilia Romagna); Center (Tuscany, Lazio); South (Abruzzo, Basilicata, Calabria, Sicily).

Table S.15. Age-standardized (European standard population) sedentariness during leisure time and at work prevalence by sex, age classes, geographical area and educational level.
Men and women residing in Italy aged 35-74 years, Health Examination Survey 2018-2019 - CUORE Project.

	Men						Women					
**Sedentariness during leisure time**												
	N	n	%	CI 95%		χ^2 p-value	N	n	%	CI 95%		χ^2 p-value
**All**	**1035**	**335**	**33,3**	**27,5**	**39,0**		**1071**	**474**	**44,5**	**38,6**	**50,5**	
**Age classes (years)**												
35-44	249	92	37,0	31,0	42,9	<0.0001	238	102	42,9	36,6	49,1	0,6034
45-54	249	101	40,6	34,5	46,7		272	130	47,8	41,9	53,7	
55-64	277	84	30,3	24,9	35,7		282	124	44,0	38,2	49,8	
65-74	260	58	22,3	17,3	27,4		279	118	42,3	36,5	48,1	
**Italian Area**												
North	410	124	30,8	21,9	39,8	0,1783	426	155	36,8	27,6	46,0	<0.0001
Center	207	62	30,8	18,2	43,4		213	91	42,8	29,4	56,2	
South	418	149	36,9	27,7	46,2		432	228	53,0	43,6	62,5	
**Education**												
Primary and middle school	304	115	39,3	28,1	50,5	<0.0001	341	182	53,5	42,6	64,4	0,5837
High school and university	728	220	30,9	24,3	37,6		728	291	40,5	33,5	47,6	
**Sedentariness at work**												
	N	n	%	CI 95%		χ^2 p-value	N	n	%	CI 95%		χ^2 p-value
**All**	**767**	**332**	**45,0**	**38,1**	**51,9**		**833,0**	**290**	**47,0**	**40,3**	**53,6**	
**Age classes (years)**												
35-44	240	89	38,4	26,6	50,1	0,0125	231	92	47,4	35,1	59,7	0,9172
45-54	244	104	44,1	32,2	56,0		265	94	45,2	33,7	56,7	
55-64	227	117	53,7	40,5	66,8		244	92	48,7	36,0	61,4	
65-74	56	22	46,8	17,7	75,9		93	12	46,2	23,4	68,9	
**Italian Area**												
North	315	140	45,5	34,7	56,3	0,6505	314	128	50,7	39,9	61,6	0,0557
Center	159	73	47,2	31,9	62,4		166	66	51,1	36,0	66,2	
South	293	119	43,2	32,1	54,3		353	96	40,6	30,5	50,7	
**Education**												
Primary and middle school	175	31	18,9	7,4	30,4	<0.0001	231	25	20,9	10,3	31,4	<0.0001
High school and university	589	301	52,3	44,4	60,2		600	265	53,1	45,3	60,9	
**Sedentariness during leisure time and at work**												
	N	n	%	CI 95%		χ^2 p-value	N	n	%	CI 95%		χ^2 p-value
**All**	**752**	**114**	**15,3**	**10,3**	**20,3**		**734**	**132**	**18,2**	**13,0**	**23,4**	
**Age classes (years)**												
35-44	234	30	12,8	4,7	20,9	0,0412	213	41	19,3	9,5	29,0	0,0940
45-54	241	47	19,5	10,0	29,0		237	44	18,6	9,6	27,5	
55-64	224	34	15,2	5,7	24,6		221	43	19,5	9,4	29,5	
65-74	53	3	5,7	0,0	19,1		63	4	6,4	0,0	17,4	
**Italian Area**												
North	312	41	13,4	6,0	20,7	0,3834	296	49	17,0	8,8	25,1	0,7056
Center	155	24	15,5	4,4	26,5		151	29	19,5	7,6	31,5	
South	285	49	17,4	8,9	25,9		287	54	18,8	10,8	26,8	
**Education**												
Primary and middle school	169	14	8,2	0,2	16,3	0,0068	172	15	8,9	1,5	16,3	0,0005
High school and university	583	100	17,4	11,4	23,4		562	117	20,9	14,6	27,3	

N: number of participants denominator.
n: number of participants related to the prevalence numerator.
Sedentariness during leisure time is defined as someone who usually carries out activities such as reading, watching television, getting to the movies or spend leisure time in other sedentary activities.
Sedentariness at work is defined as one whose work mainly involves sitting at a desk and generally without the need to walk.
SD: standard deviation; CI: confidence interval.
Prevalence was age standardized by European standard population distribution 2013 (except when it is reported by age-classes).
Chi-square test to compare prevalence among classes.
Pool of the following Italian regions: Piedmont, Lombardy, Liguria, Emilia Romagna, Tuscany, Lazio, Abruzzo, Basilicata, Calabria, and Sicily.
Italian Area: North (Piedmont, Lombardy, Liguria, Emilia Romagna); Center (Tuscany, Lazio); South (Abruzzo, Basilicata, Calabria, Sicily).

Table S.16. Age-standardized (European standard population) balanced nutrition food groups intake (EPIC questionnaire): vegetables, fruit, fish and cheese prevalence by sex, age classes, geographical area and educational level.
Men and women residing in Italy aged 35-74 years, Health Examination Survey 2018-2019 - CUORE Project.

	Men						Women					
**Vegetables**												
	N	n	%	CI 95%		χ^2 p-value	N	n	%	CI 95%		χ^2 p-value
**All**	**793**	**222**	**28,1**	**21,9**	**34,4**		**785**	**302**	**39,0**	**32,2**	**45,8**	
**Age classes (years)**												
35-44	196	51	26,0	19,9	32,2	0,4916	191	86	45,0	38,0	52,1	0,0301
45-54	204	61	29,9	23,6	36,2		216	90	41,7	35,1	48,2	
55-64	219	67	30,6	24,5	36,7		214	74	34,6	28,2	41,0	
65-74	174	43	24,7	18,3	31,1		164	52	31,7	24,6	38,8	
**Italian Area**												
North	357	97	27,2	18,0	36,5	0,2484	359	140	39,6	29,5	49,7	0,2616
Center	180	59	32,7	19,0	46,5		184	78	43,4	29,0	57,8	
South	256	66	26,1	15,4	36,9		242	84	34,8	22,9	46,7	
**Education**												
Primary and middle school	201	50	24,7	12,6	36,8	0,2883	209	68	33,0	20,0	45,9	0,0463
High school and university	591	172	29,3	22,0	36,6		575	234	41,1	33,2	49,1	
**Fruit**												
	N	n	%	CI 95%		χ^2 p-value	N	n	%	CI 95%		χ^2 p-value
**All**	**793**	**394**	**49,7**	**42,7**	**56,6**		**785**	**411**	**52,3**	**45,3**	**59,2**	
**Age classes (years)**												
35-44	196	96	49,0	42,0	56,0	0,6598	191	98	51,3	44,2	58,4	0,9483
45-54	204	106	52,0	45,1	58,8		216	111	51,4	44,7	58,1	
55-64	219	102	46,6	40,0	53,2		214	115	53,7	47,1	60,4	
65-74	174	90	51,7	44,3	59,2		164	87	53,1	45,4	60,7	
**Italian Area**												
North	357	179	50,0	39,6	60,4	0,2694	359	196	55,4	45,1	65,7	0,4259
Center	180	97	53,6	39,0	68,2		184	96	51,4	36,9	65,9	
South	256	118	46,5	34,3	58,7		242	119	48,4	35,9	60,9	
**Education**												
Primary and middle school	201	104	51,5	37,4	65,5	0,5390	209	111	53,2	39,4	67,0	0,8798
High school and university	591	289	49,0	41,0	57,0		575	300	52,1	44,0	60,1	
**Vegetable and Fruit**												
	N	n	%	CI 95%		χ^2 p-value	N	n	%	CI 95%		χ^2 p-value
**All**	**793**	**104**	**13,3**	**8,6**	**18,1**		**785**	**154**	**19,9**	**14,3**	**25,4**	
**Age classes (years)**												
35-44	196	26	13,3	4,2	22,4	0,2520	191	39	20,4	9,5	31,4	0,3179
45-54	204	34	16,7	6,9	26,5		216	50	23,2	12,4	33,9	
55-64	219	27	12,3	3,5	21,1		214	39	18,2	7,8	28,7	
65-74	174	17	9,8	0,0	19,5		164	26	15,9	3,5	28,2	
**Italian Area**												
North	357	50	14,1	6,9	21,3	0,0678	359	82	23,4	14,7	32,2	0,1014
Center	180	30	16,9	5,9	27,9		184	33	18,2	7,0	29,4	
South	256	24	9,9	2,6	17,1		242	39	16,0	6,8	25,1	
**Education**												
Primary and middle school	201	22	10,9	2,2	19,7	0,3465	209	35	17,3	6,9	27,7	0,2589
High school and university	591	82	14,2	8,6	19,7		575	119	20,8	14,2	27,4	
**Fish**												
	N	n	%	CI 95%		χ^2 p-value	N	n	%	CI 95%		χ^2 p-value
**All**	**793**	**350**	**44,4**	**37,5**	**51,3**		**785**	**319**	**40,5**	**33,6**	**47,3**	
**Age classes (years)**												
35-44	196	93	47,5	40,5	54,4	0,4272	191	77	40,3	33,4	47,3	0,6847
45-54	204	90	44,1	37,3	50,9		216	81	37,5	31,0	44,0	
55-64	219	99	45,2	38,6	51,8		214	91	42,5	35,9	49,2	
65-74	174	68	39,1	31,8	46,3		164	70	42,7	35,1	50,3	
**Italian Area**												
North	357	165	46,9	36,6	57,2	0,5635	359	144	39,4	29,3	49,5	0,8432
Center	180	76	42,0	27,5	56,4		184	73	39,8	25,6	54,1	
South	256	109	42,7	30,6	54,7		242	102	42,4	30,1	54,8	
**Education**												
Primary and middle school	201	79	40,0	26,3	53,8	0,1252	209	84	39,7	26,2	53,2	0,9293
High school and university	591	271	45,9	37,9	53,9		575	235	40,8	32,9	48,8	
**Cheese**												
	N	n	%	CI 95%		χ^2 p-value	N	n	%	CI 95%		χ^2 p-value
**All**	**793**	**355**	**44,7**	**37,8**	**51,6**		**785**	**421**	**53,5**	**46,6**	**60,5**	
**Age classes (years)**												
35-44	196	90	45,9	38,9	52,9	0,7559	191	96	50,3	43,2	57,4	0,7558
45-54	204	85	41,7	34,9	48,4		216	119	55,1	48,5	61,7	
55-64	219	102	46,6	40,0	53,2		214	116	54,2	47,5	60,9	
65-74	174	78	44,8	37,4	52,2		164	90	54,9	47,3	62,5	
**Italian Area**												
North	357	149	41,8	31,6	52,0	0,2765	359	189	52,9	42,6	63,2	0,6255
Center	180	83	45,7	31,1	60,3		184	96	51,7	37,2	66,2	
South	256	123	48,0	35,8	60,2		242	136	55,9	43,5	68,3	
**Education**												
Primary and middle school	201	87	43,3	29,3	57,2	0,7006	209	123	58,7	45,1	72,3	0,0962
High school and university	591	267	45,1	37,1	53,0		575	298	51,9	43,8	60,0	

N: number of participants denominator.
n: number of participants related to the prevalence numerator.
For vegetables intake, the following foods were considered: leafy vegetables-cooked, leafy vegetables-raw, other vegetables, tomatoes-raw, tomatoes-cooked, root vegetables, cabbages, mushrooms, grain and pod vegetables, onion, garlic, stalk vegetables, mixed salad, mixed vegetables.
For vegetables intake were considered the following foods: leafy vegetable-cooked, leafy vegetable-raw, other vegetables, tomatoes-raw, tomatoes-cooked, root vegetables, cabbages, mushrooms, grain and pod vegetables, onion, garlic, stalk vegetables, mixed salad, mixed vegetables.
For fruits intake, the following foods were considered: citrus fruits and other fruits.
For fish intake, the following foods were considered: fish, crustaceans and molluscs.
For cheese intake, the following foods were considered: cheeses (including fresh cheeses).
Dietary information was collected by the self-administered Italian version of the food frequency questionnaire (FFQ) of the European Prospective Investigation into Cancer (EPIC).
A balanced nutrition was defined based on the following intake: vegetables ≥200 g/day; fruit 150-375 g/day; fish at least twice per week (150 g per serving); cheese no more than three times per week (50-100 g per serving); sausages, salami and other preserved meat no more than once per week (50 g per serving); cake and desserts no more than once per week (100 g per serving); sugar beverages less than one per week (330 ml); and consumption of alcoholic beverages limited to two glasses per day for men (24 g of ethanol), one glass per day for women (12 g of ethanol) and one glass per day for men and women aged 65-74 (12 g of ethanol).
SD: standard deviation; CI: confidence interval.
Prevalence was age standardized by European standard population distribution 2013 (except when it is reported by age-classes).
Chi-square test to compare prevalence among classes.

Pool of the following Italian regions: Piedmont, Lombardy, Liguria, Emilia Romagna, Tuscany, Lazio, Basilicata, Calabria, and Sicily.
Italian Area: North (Piedmont, Lombardy, Liguria, Emilia Romagna); Center (Tuscany, Lazio); South (Basilicata, Calabria, Sicily).

Table S.17. Age-standardized (European standard population) balanced nutrition food groups intake (EPIC questionnaire): processed meat, sweets/cakes, sweet drinks and alcohol prevalence by sex, age classes, geographical area and educational level.

Men and women residing in Italy aged 35-74 years, Health Examination Survey 2018-2019 - CUORE Project.

	Men						Women					
**Processed meat**												
	N	n	%	CI 95%		χ^2 p-value	N	n	%	CI 95%		χ^2 p-value
**All**	**793**	**91**	**11,1**	**6,8**	**15,5**		**785**	**168**	**20,7**	**15,0**	**26,3**	
**Age classes (years)**												
35-44	196	12	6,1	2,8	9,5	0,0146	191	28	14,7	9,6	19,7	<0.0001
45-54	204	21	10,3	6,1	14,5		216	35	16,2	11,3	21,1	
55-64	219	30	13,7	9,1	18,3		214	52	24,3	18,6	30,1	
65-74	174	28	16,1	10,6	21,6		164	53	32,3	25,2	39,5	
**Italian Area**												
North	357	37	10,1	3,9	16,3	0,1479	359	83	22,1	13,6	30,7	0,4115
Center	180	28	15,1	4,6	25,6		184	40	21,4	9,5	33,3	
South	256	26	9,8	2,6	17,1		242	45	18,1	8,5	27,7	
**Education**												
Primary and middle school	201	16	7,6	0,2	15,1	0,0913	209	54	24,5	12,6	36,4	0,0863
High school and university	591	75	12,3	7,0	17,6		575	114	19,4	13,0	25,8	
**Sweets/Cakes**												
	N	n	%	CI 95%		χ^2 p-value	N	n	%	CI 95%		χ^2 p-value
**All**	**793**	**74**	**9,0**	**5,0**	**13,0**		**785**	**87**	**10,7**	**6,4**	**15,0**	
**Age classes (years)**												
35-44	196	11	5,6	2,4	8,8	0,0195	191	12	6,3	2,8	9,7	0,0051
45-54	204	14	6,9	3,4	10,3		216	18	8,3	4,7	12,0	
55-64	219	25	11,4	7,2	15,6		214	30	14,0	9,4	18,7	
65-74	174	24	13,8	8,7	18,9		164	27	16,5	10,8	22,1	
**Italian Area**												
North	357	29	8,0	2,4	13,6	0,1003	359	37	9,8	3,7	15,9	0,2929
Center	180	13	6,9	0,0	14,3		184	17	8,7	0,5	16,9	
South	256	32	11,9	4,0	19,8		242	33	13,5	5,0	22,0	
**Education**												
Primary and middle school	201	14	6,9	0,0	14,0	0,2298	209	26	11,8	2,9	20,7	0,5529
High school and university	591	60	9,7	0,0	14,5		575	61	10,3	5,4	15,2	
**Sweet Drinks**												
	N	n	%	CI 95%		χ^2 p-value	N	n	%	CI 95%		χ^2 p-value
**All**	**793**	**432**	**53,8**	**46,8**	**60,7**		**785**	**473**	**59,3**	**52,4**	**66,1**	
**Age classes (years)**												
35-44	196	81	41,3	34,4	48,2	<0.0001	191	87	45,6	38,5	52,6	<0.0001
45-54	204	108	52,9	46,1	59,8		216	123	56,9	50,3	63,6	
55-64	219	133	60,7	54,3	67,2		214	141	65,9	59,5	72,2	
65-74	174	110	63,2	56,1	70,4		164	122	74,4	67,7	81,1	
**Italian Area**												
North	357	194	53,6	43,3	64,0	0,8149	359	216	59,1	48,9	69,2	0,8341
Center	180	95	52,3	37,7	67,0		184	108	57,6	43,2	71,9	
South	256	143	54,9	42,8	67,1		242	149	60,9	48,7	73,1	
**Education**												
Primary and middle school	201		51,4	37,4	65,5	0,6365	209		61,7	48,3	75,1	0,4669
High school and university	591		54,4	46,5	62,4		575		58,6	50,6	66,5	
**Alcohol**												
	N	n	%	CI 95%		χ^2 p-value	N	n	%	CI 95%		χ^2 p-value
**All**	**793**	**581**	**74,2**	**68,1**	**80,3**		**785**	**662**	**84,4**	**79,4**	**89,5**	
**Age classes (years)**												
35-44	196	162	82,7	72,5	92,8	<0.0001	191	165	86,4	77,1	95,7	0,824
45-54	204	155	76,0	64,8	87,2		216	182	84,3	75,0	93,6	
55-64	219	167	76,3	64,9	87,7		214	178	83,2	73,0	93,3	
65-74	174	97	55,8	39,4	72,1		164	137	83,5	71,0	96,1	
**Italian Area**												
North	357	248	70,4	61,0	79,9	0,0438	359	292	81,6	73,6	89,6	0,0198
Center	180	143	80,4	68,8	92,1		184	153	83,0	72,0	93,9	
South	256	190	75,2	64,6	85,7		242	217	89,6	82,0	97,3	
**Education**												
Primary and middle school	201	132	66,5	53,3	79,8	0,0058	209	182	86,7	77,3	96,0	0,2401
High school and university	591	449	76,9	70,1	83,6		575	479	83,6	77,6	89,6	

N: number of participants denominator.
n: number of participants related to the prevalence numerator.
For processed meat intake, the following foods were considered: sausages, salami and other preserved meat.
For sweets/cakes intake, the following foods were considered: sugar, honey, jam, chocolate, candy bars, paste, confetti/flakes, confectionery non chocolate, ice cream, cakes, pies, pastries, puddings (not milk based), dry cakes, biscuits.
For sweet beverages intake, the following foods were considered: fruit and vegetable juices, carbonated/soft/isotonic drinks, diluted syrups.
For alcohol intake, the following foods were considered: alcoholic beverages.
Dietary information was collected by the self-administered Italian version of the food frequency questionnaire (FFQ) of the European Prospective Investigation into Cancer (EPIC).
A balanced nutrition was defined based on the following intake: vegetables ≥200 g/day; fruit 150-375 g/day; fish at least twice per week (150 g per serving); cheese no more than three times per week (50-100 g per serving); sausages, salami and other preserved meat no more than once per week (50 g per serving); cake and desserts no more than once per week (100 g per serving); sugar beverages less than one per week (330 ml); and consumption of alcoholic beverages limited to two glasses per day for men (24 g of ethanol), one glass per day for women (12 g of ethanol) and one glass per day for men and women aged 65-74 (12 g of ethanol).
SD: standard deviation; CI: confidence interval.
Prevalence was age standardized by European standard population distribution 2013 (except when it is reported by age-classes).
Chi-square test to compare prevalence among classes.

Pool of the following Italian regions: Piedmont, Lombardy, Liguria, Emilia Romagna, Tuscany, Lazio, Basilicata, Calabria, and Sicily.
Italian Area: North (Piedmont, Lombardy, Liguria, Emilia Romagna); Center (Tuscany, Lazio); South (Basilicata, Calabria, Sicily).

Table S.18. Age-standardized (European standard population) food group intake (EPIC questionnaire): vegetables, fruit, fish and cheese mean by sex, age classes, geographical area and educational level. Men and women residing in Italy aged 35-74 years, Health Examination Survey 2018-2019 - CUORE Project.

	Men						Women					
**Vegetable (g/day)**												
	N	Mean	SD	CI 95%		anova p-value	N	Mean	SD	CI 95%		anova p-value
**All**	**793**	**167,9**	**93,2**	**161,4**	**174,4**		**785**	**184,9**	**96,8**	**178,2**	**191,7**	
**Age classes (years)**												
35-44	196	163,1	84,2	151,3	174,9	0,0031	191	202,1	104,2	187,3	216,9	0,0031
45-54	204	179,4	106,0	164,9	194,0		216	188,3	90,5	176,2	200,4	
55-64	219	169,2	91,3	157,1	181,3		214	175,7	91,5	163,5	188,0	
65-74	174	155,3	86,0	142,6	168,1		164	166,6	99,9	151,3	181,9	
**Italian Area**												
North	357	166,8	91,7	157,3	176,3	0,0010	359	184,4	100,3	174,0	194,8	0,0010
Center	180	182,2	88,4	169,3	195,1		184	200,2	98,2	186,0	214,4	
South	256	159,6	97,5	147,6	171,5		242	174,4	89,2	163,2	185,7	
**Education**												
Primary and middle school	201	156,1	79,6	145,1	167,1	<0,0001	209	163,3	85,9	151,6	174,9	<0,0001
High school and university	591	172,0	96,9	164,2	179,8		575	192,4	99,4	184,3	200,5	
**Fruit (g/day)**												
	N	Mean	SD	CI 95%		anova p-value	N	Mean	SD	CI 95%		anova p-value
**All**	**793**	**267,9**	**174,9**	**255,7**	**280,1**		**785**	**275,7**	**164,4**	**264,2**	**287,2**	
**Age classes (years)**												
35-44	196	243,9	175,1	219,3	268,4	0,0013	191	270,6	145,2	250,0	291,2	0,0013
45-54	204	257,3	165,5	234,6	280,0		216	255,5	164,2	233,6	277,4	
55-64	219	290,1	183,2	265,8	314,4		214	294,2	166,7	271,9	316,5	
65-74	174	287,2	171,7	261,7	312,7		164	290,3	185,3	262,0	318,7	
**Italian Area**												
North	357	255,4	168,6	237,9	272,9	0,4007	359	276,1	162,3	259,3	292,9	0,4007
Center	180	264,2	170,4	239,3	289,1		184	295,5	172,4	270,6	320,4	
South	256	287,7	185,2	265,0	310,4		242	260,4	160,6	240,2	280,7	
**Education**												
Primary and middle school	201	272,8	172,7	248,9	296,7	0,9245	209	268,6	157,2	247,3	289,9	0,9245
High school and university	591	266,4	175,9	252,2	280,6		575	277,9	167,0	264,2	291,5	
**Vegetable and Fruit (g/day)**												
	N	Mean	SD	CI 95%		anova p-value	N	Mean	SD	CI 95%		anova p-value
**All**	**793**	**435,8**	**221,2**	**420,4**	**451,2**		**785**	**460,6**	**213,8**	**445,7**	**475,6**	
**Age classes (years)**												
35-44	196	407,0	219,1	376,3	437,7	0,3103	191	472,7	205,0	443,6	501,8	0,3103
45-54	204	436,7	209,3	408,0	465,4		216	443,8	208,7	416,0	471,6	
55-64	219	459,2	230,5	428,7	489,8		214	469,9	216,3	440,9	498,9	
65-74	174	442,5	225,6	409,0	476,1		164	457,0	231,8	421,5	492,4	
**Italian Area**												
North	357	422,2	218,6	399,5	444,9	0,0692	359	460,5	213,4	438,4	482,5	0,0692
Center	180	446,4	210,5	415,7	477,2		184	495,7	226,1	463,0	528,4	
South	256	447,3	231,5	418,9	475,6		242	434,9	202,0	409,4	460,3	
**Education**												
Primary and middle school	201	428,9	208,9	400,0	457,8	0,0654	209	431,9	207,3	403,8	460,0	0,0654
High school and university	591	438,4	225,2	420,2	456,5		575	470,3	215,4	452,7	487,9	
**Fish (g/day)**												
	N	Mean	SD	CI 95%		anova p-value	N	Mean	SD	CI 95%		anova p-value
**All**	**793**	**45,5**	**34,7**	**43,1**	**47,9**		**785**	**42,7**	**30,5**	**40,5**	**44,8**	
**Age classes (years)**												
35-44	196	46,6	35,1	41,7	51,6	0,6242	191	42,2	25,7	38,5	45,8	0,6242
45-54	204	45,3	33,7	40,6	49,9		216	42,1	33,0	37,7	46,5	
55-64	219	47,0	36,0	42,3	51,8		214	43,8	31,6	39,5	48,0	
65-74	174	42,0	33,9	37,0	47,0		164	42,8	31,3	38,0	47,6	
**Italian Area**												
North	357	47,7	36,7	43,9	51,5	0,3656	359	42,6	29,2	39,6	45,6	0,3656
Center	180	41,3	28,7	37,1	45,5		184	43,2	32,6	38,5	47,9	
South	256	45,4	35,6	41,0	49,7		242	42,4	30,7	38,5	46,2	
**Education**												
Primary and middle school	201	42,1	33,5	37,5	46,8	0,0504	209	41,0	30,5	36,9	45,2	0,0504
High school and university	591	46,7	35,1	43,8	49,5		575	43,3	30,4	40,8	45,8	
**Cheese (g/day)**												
	N	Mean	SD	CI 95%		anova p-value	N	Mean	SD	CI 95%		anova p-value
**All**	**793**	**42,7**	**34,3**	**40,4**	**45,1**		**785**	**37,2**	**31,5**	**35,0**	**39,4**	
**Age classes (years)**												
35-44	196	44,7	37,8	39,4	50,0	0,0997	191	41,2	36,2	36,1	46,4	0,0997
45-54	204	43,0	33,6	38,4	47,6		216	36,7	28,1	33,0	40,4	
55-64	219	42,4	33,1	38,0	46,8		214	35,6	29,5	31,7	39,6	
65-74	174	39,9	31,6	35,2	44,6		164	34,0	31,8	29,2	38,9	
**Italian Area**												
North	357	46,5	36,8	42,7	50,3	0,0169	359	38,5	33,6	35,1	42,0	0,0169
Center	180	38,5	30,4	34,0	42,9		184	37,2	29,0	33,0	41,3	
South	256	40,5	32,7	36,5	44,6		242	35,2	30,0	31,4	38,9	
**Education**												
Primary and middle school	201	42,8	34,6	38,0	47,5	0,2342	209	33,9	33,4	29,4	38,4	0,2342
High school and university	591	42,8	34,2	40,0	45,5		575	38,3	30,8	35,8	40,8	

For vegetables intake, the following foods were considered: leafy vegetables-cooked, leafy vegetables-raw, other vegetables, tomatoes-raw, tomatoes-cooked, root vegetables, cabbages, mushrooms, grain and pod vegetables, onion, garlic, stalk vegetables, mixed salad, mixed vegetables.
For vegetables intake were considered the following foods: leafy vegetable-cooked, leafy vegetable-raw, other vegetables, tomatoes-raw, tomatoes-cooked, root vegetables, cabbages, mushrooms, grain and pod vegetables, onion, garlic, stalk vegetables, mixed salad, mixed vegetables.
For fruits intake, the following foods were considered: citrus fruits and other fruits.
For fish intake, the following foods were considered: fish, crustaceans and molluscs.
For cheese intake, the following foods were considered: cheeses (including fresh cheeses).
Dietary information was collected by the self-administered Italian version of the food frequency questionnaire (FFQ) of the European Prospective Investigation into Cancer (EPIC).
SD: standard deviation; CI: confidence interval.
Means and standard deviations were age standardized by European standard population distribution 2013 (except when they are reported by age-classes).
Anova to compare mean values among classes.
Pool of the following Italian regions: Piedmont, Lombardy, Liguria, Emilia Romagna, Tuscany, Lazio, Basilicata, Calabria, and Sicily.
Italian Area: North (Piedmont, Lombardy, Liguria, Emilia Romagna); Center (Tuscany, Lazio); South (Basilicata, Calabria, Sicily).

Table S.19. Age-standardized (European standard population) food group intake (EPIC questionnaire): processed meat, sweets/cakes, sweet drinks and alcohol mean by sex, age classes, geographical area and educational level. Men and women residing in Italy aged 35-74 years, Health Examination Survey 2018-2019 - CUORE Project.

	Men						Women					
**Processed meat (g/day)**												
	**N**	**Mean**	**SD**	**CI 95%**		**anova p-value**	**N**	**Mean**	**SD**	**CI 95%**		**anova p-value**
**All**	**793**	**36,4**	**28,8**	**34,4**	**38,4**		**785**	**24,6**	**21,7**	**23,0**	**26,1**	
**Age classes (years)**												
35-44	196	44,9	32,0	40,4	49,3	<0,0001	191	29,1	23,2	25,8	32,4	<0,0001
45-54	204	39,5	30,4	35,3	43,6		216	27,7	21,7	24,8	30,6	
55-64	219	32,8	25,9	29,4	36,3		214	21,2	21,4	18,3	24,1	
65-74	174	24,1	18,8	21,3	26,9		164	17,2	16,5	14,7	19,8	
**Italian Area**												
North	357	33,3	25,6	30,7	36,0	0,0011	359	23,9	21,4	21,6	26,1	0,0011
Center	180	34,9	27,1	30,9	38,8		184	23,8	21,5	20,7	26,9	
South	256	41,7	33,1	37,6	45,8		242	26,1	22,3	23,3	28,9	
**Education**												
Primary and middle school	201	39,8	28,1	35,9	43,7	0,5038	209	23,3	22,2	20,3	26,3	0,5038
High school and university	591	35,3	29,0	32,9	37,6		575	24,9	21,3	23,1	26,6	
**Sweets/Cakes (g/day)**												
	**N**	**Mean**	**SD**	**CI 95%**		**anova p-value**	**N**	**Mean**	**SD**	**CI 95%**		**anova p-value**
**All**	**793**	**83,9**	**59,6**	**79,7**	**88,0**		**785**	**77,8**	**61,8**	**73,4**	**82,1**	
**Age classes (years)**												
35-44	196	89,5	58,3	81,4	97,7	<0,0001	191	87,1	63,1	78,2	96,1	<0,0001
45-54	204	93,0	64,3	84,1	101,8		216	85,3	70,3	75,9	94,7	
55-64	219	79,9	57,1	72,4	87,5		214	70,8	54,9	63,4	78,1	
65-74	174	67,2	54,0	59,2	75,2		164	60,8	48,0	53,5	68,2	
**Italian Area**												
North	357	87,4	60,5	81,1	93,7	0,1249	359	80,3	59,1	74,2	86,4	0,1249
Center	180	78,6	53,5	70,7	86,4		184	73,7	59,4	65,1	82,3	
South	256	82,8	62,2	75,1	90,4		242	77,0	67,1	68,6	85,5	
**Education**												
Primary and middle school	201	90,2	68,5	80,7	99,7	0,8558	209	73,6	58,8	65,7	81,6	0,8558
High school and university	591	81,6	56,0	77,0	86,1		575	79,1	62,8	73,9	84,2	
**Sweet Drinks (ml/day)**												
	**N**	**Mean**	**SD**	**CI 95%**		**anova p-value**	**N**	**Mean**	**SD**	**CI 95%**		**anova p-value**
**All**	**793**	**99,9**	**159,9**	**88,8**	**111,1**		**785**	**73,9**	**131,4**	**64,7**	**83,1**	
**Age classes (years)**												
35-44	196	124,7	177,9	99,8	149,6	<0.0001	191	108,8	158,9	86,2	131,3	<0.0001
45-54	204	111,2	170,6	87,7	134,6		216	75,4	131,1	57,9	92,9	
55-64	219	81,8	133,7	64,1	99,5		214	59,4	121,0	43,2	75,6	
65-74	174	72,5	143,4	51,2	93,8		164	39,7	77,7	27,8	51,6	
**Italian Area**												
North	357	93,3	139,3	78,9	107,8	0,1505	359	74,8	130,8	61,3	88,3	0,1505
Center	180	83,3	132,6	64,0	102,7		184	73,4	127,7	54,9	91,8	
South	256	120,7	197,9	96,4	144,9		242	73,0	135,5	56,0	90,1	
**Education**												
Primary and middle school	201	131,9	208,6	103,1	160,7	0,1004	209	65,8	116,6	50,0	81,7	0,1004
High school and university	591	89,7	139,2	78,5	100,9		575	76,5	136,2	65,4	87,7	
**Alcohol (g/day)**												
	**N**	**Mean**	**SD**	**CI 95%**		**anova p-value**	**N**	**Mean**	**SD**	**CI 95%**		**anova p-value**
**All**	**793**	**14,67**	**16,33**	**13,53**	**15,8**		**785**	**5,53**	**9,19**	**4,89**	**6,18**	
**Age classes (years)**												
35-44	196	13,7	14,8	11,6	15,8	0,7067	191	6,1	10,4	4,6	7,6	0,7067
45-54	204	14,9	16,8	12,6	17,2		216	5,1	8,6	4,0	6,3	
55-64	219	14,4	17,0	12,2	16,7		214	5,4	8,5	4,3	6,6	
65-74	174	16,1	16,9	13,6	18,6		164	5,5	9,3	4,1	6,9	
**Italian Area**												
North	357	16,1	16,0	14,5	17,8	0,0037	359	6,8	10,5	5,7	7,8	0,0037
Center	180	12,2	14,2	10,1	14,3		184	5,9	8,4	4,7	7,1	
South	256	14,4	17,9	12,2	16,6		242	3,5	7,2	2,6	4,4	
**Education**												
Primary and middle school	201	17,2	27,5	13,4	21,0	0,7387	209	5,2	9,0	4,0	6,4	0,7387
High school and university	591	15,3	20,0	13,7	16,9		575	5,6	11,1	4,7	6,5	

For processed meat intake, the following foods were considered: sausages, salami and other preserved meat.
For sweets/cakes intake, the following foods were considered: sugar, honey, jam, chocolate, candy bars, paste, confetti/flakes, confectionery non chocolate, ice cream, cakes, pies, pastries, puddings (not milk based), dry cakes, biscuits.
For sweet beverages intake, the following foods were considered: fruit and vegetable juices, carbonated/soft/isotonic drinks, diluted syrups.
For alcohol intake, the following foods were considered: alcoholic beverages.
Dietary information was collected by the self-administered Italian version of the food frequency questionnaire (FFQ) of the European Prospective Investigation into Cancer (EPIC).
SD: standard deviation; CI: confidence interval.
Means and standard deviations were age standardized by European standard population distribution 2013 (except when they are reported by age-classes).
Anova to compare mean values among classes.
Pool of the following Italian regions: Piedmont, Lombardy, Liguria, Emilia Romagna, Tuscany, Lazio, Basilicata, Calabria, and Sicily.
Italian Area: North (Piedmont, Lombardy, Liguria, Emilia Romagna); Center (Tuscany, Lazio); South (Basilicata, Calabria, Sicily).

Table S.20. Age-standardized (European standard population) food groups intake (EPIC questionnaire): cereals, potatoes, legumes and oil mean by sex, age classes, geographical area and educational level. Men and women residing in Italy aged 35-74 years, Health Examination Survey 2018-2019 - CUORE Project.

	Men						Women					
**Cereals (g/day)**												
	**N**	**Mean**	**SD**	**CI 95%**		**anova p-value**	**N**	**Mean**	**SD**	**CI 95%**		**anova p-value**
**All**	**793**	**177,2**	**95,7**	**170,6**	**183,9**		**785**	**135,9**	**84,8**	**130,0**	**141,8**	
**Age classes (years)**												
35-44	196	176,6	96,9	163,0	190,2	0,1230	191	138,9	84,4	127,0	150,9	0,1230
45-54	204	169,8	89,0	157,5	182,0		216	138,9	89,1	127,0	150,8	
55-64	219	190,3	102,5	176,7	203,9		214	136,6	83,4	125,4	147,8	
65-74	174	170,2	92,1	156,6	183,9		164	124,9	79,7	112,7	137,1	
**Italian Area**												
North	357	181,5	99,9	171,2	191,9	0,5273	359	131,7	85,5	122,9	140,6	0,5273
Center	180	177,0	88,3	164,1	189,9		184	144,8	81,8	133,0	156,7	
South	256	171,4	94,6	159,8	183,0		242	135,4	85,9	124,6	146,2	
**Education**												
Primary and middle school	201	178,2	91,6	165,6	190,9	0,0375	209	119,8	73,8	109,8	129,8	0,0375
High school and university	591	176,8	97,1	169,0	184,6		575	141,3	87,7	134,1	148,4	
**Potatoes (g/day)**												
	**N**	**Mean**	**SD**	**CI 95%**		**anova p-value**	**N**	**Mean**	**SD**	**CI 95%**		**anova p-value**
**All**	**793**	**22,9**	**19,9**	**21,5**	**24,3**		**785**	**21,2**	**22,7**	**19,6**	**22,7**	
**Age classes (years)**												
35-44	196	25,8	19,1	23,1	28,4	<0,0001	191	25,0	29,5	20,8	29,2	<0,0001
45-54	204	24,9	19,5	22,2	27,6		216	24,0	21,4	21,2	26,9	
55-64	219	21,0	20,7	18,3	23,8		214	17,4	17,8	15,1	19,8	
65-74	174	18,2	19,4	15,4	21,1		164	15,9	17,3	13,3	18,6	
**Italian Area**												
North	357	22,4	20,2	20,3	24,5	0,0265	359	19,5	18,5	17,6	21,4	0,0265
Center	180	23,3	20,3	20,4	26,3		184	19,5	19,8	16,7	22,4	
South	256	23,3	19,2	21,0	25,7		242	24,8	29,0	21,1	28,4	
**Education**												
Primary and middle school	201	21,3	19,6	18,6	24,0	0,1830	209	20,5	20,8	17,7	23,3	0,1830
High school and university	591	23,4	19,9	21,7	25,0		575	21,4	23,3	19,5	23,3	
**Legumes (g/day)**												
	**N**	**Mean**	**SD**	**CI 95%**		**anova p-value**	**N**	**Mean**	**SD**	**CI 95%**		**anova p-value**
**All**	**793**	**21,6**	**19,0**	**20,3**	**23,0**		**785**	**19,7**	**16,0**	**18,6**	**20,8**	
**Age classes (years)**												
35-44	196	22,9	21,9	19,8	26,0	0,0710	191	21,0	17,9	18,4	23,5	0,0710
45-54	204	21,9	18,0	19,5	24,4		216	20,1	15,5	18,1	22,2	
55-64	219	21,3	18,8	18,8	23,8		214	19,4	15,4	17,3	21,5	
65-74	174	19,7	16,0	17,3	22,1		164	17,5	14,1	15,3	19,6	
**Italian Area**												
North	357	17,9	17,2	16,1	19,7	<0.0001	359	16,3	14,0	14,8	17,7	<0.0001
Center	180	22,8	19,2	20,0	25,6		184	19,8	16,8	17,3	22,2	
South	256	25,9	20,2	23,5	28,4		242	24,7	16,8	22,6	26,8	
**Education**												
Primary and middle school	201	18,8	14,2	16,9	20,8	0,0069	209	18,3	14,9	16,3	20,3	0,0069
High school and university	591	22,6	20,3	20,9	24,2		575	20,2	16,3	18,8	21,5	
**Oil (g/day)**												
	**N**	**Mean**	**SD**	**CI 95%**		**anova p-value**	**N**	**Mean**	**SD**	**CI 95%**		**anova p-value**
**All**	**793**	**15,8**	**11,34**	**15,01**	**16,59**		**785**	**17,45**	**11,3**	**16,66**	**18,24**	
**Age classes (years)**												
35-44	196	13,7	10,1	12,3	15,1	0,2151	191	19,0	11,8	17,3	20,6	0,2151
45-54	204	16,7	12,3	15,0	18,4		216	17,4	11,0	15,9	18,8	
55-64	219	17,6	11,4	16,1	19,1		214	16,7	10,9	15,2	18,2	
65-74	174	14,9	11,1	13,2	16,5		164	16,4	11,4	14,7	18,2	
**Italian Area**												
North	357	16,32	11,43	15,13	17,51	0,2149	359	17,43	11,6	16,23	18,63	0,2149
Center	180	18,1	10,91	16,51	19,7		184	20,47	11,2	18,86	22,08	
South	256	13,47	11,13	12,11	14,84		242	15,24	10,3	13,93	16,54	
**Education**												
Primary and middle school	201	13,8	11,1	12,3	15,4	<0,0001	209	14,0	10,1	12,6	15,3	<0,0001
High school and university	591	16,5	11,3	15,6	17,4		575	18,7	11,4	17,8	19,6	

For cereals intake, the following foods were considered: pasta, rice, white and whole meal bread, other grains, crispbread, rusks, and breakfast cereals.
For potatoes intake, the following foods were considered: french fries, boiled potatoes, roasted potatoes, pure potatoes, croquette potatoes.
Dietary information was collected by the self-administered Italian version of the food frequency questionnaire (FFQ) of the European Prospective Investigation into Cancer (EPIC).
SD: standard deviation; CI: confidence interval.
Means and standard deviations were age standardized by European standard population distribution 2013 (except when they are reported by age-classes).
Anova to compare mean values among classes.
Pool of the following Italian regions: Piedmont, Lombardy, Liguria, Emilia Romagna, Tuscany, Lazio, Basilicata, Calabria, and Sicily.
Italian Area: North (Piedmont, Lombardy, Liguria, Emilia Romagna); Center (Tuscany, Lazio); South (Basilicata, Calabria, Sicily).

Table S.21. Age-standardized (European standard population) food groups intake (EPIC questionnaire): meat, eggs, milk mean by sex, age classes, geographical area and educational level.
Men and women residing in Italy aged 35-74 years, Health Examination Survey 2018-2019 - CUORE Project.

	Men						Women					
**Meat (g/day)**												
	N	Mean	SD	CI 95%		anova p-value	N	Mean	SD	CI 95%		anova p-value
**All**	**793**	**94,5**	**57,7**	**90,4**	**98,5**		**785**	**80,7**	**50,3**	**77,2**	**84,2**	
**Age classes (years)**												
35-44	196	104,2	57,7	96,2	112,3	<0,0001	191	88,7	53,7	81,1	96,3	<0.0001
45-54	204	100,4	57,9	92,4	108,3		216	87,7	51,2	80,9	94,6	
55-64	219	91,7	57,9	84,0	99,3		214	70,6	46,3	64,4	76,8	
65-74	174	74,7	52,5	66,9	82,5		164	71,5	45,0	64,6	78,4	
**Italian Area**												
North	357	93,7	57,4	87,7	99,7	0,7367	359	81,2	52,9	75,7	86,7	0,7367
Center	180	94,2	55,1	86,2	102,3		184	83,9	48,9	76,8	90,9	
South	256	95,7	60,1	88,4	103,1		242	77,6	47,3	71,6	83,5	
**Education**												
Primary and middle school	201	98,9	58,3	90,9	107,0	0,6666	209	79,4	51,7	72,4	86,4	0,6666
High school and university	591	93,0	57,6	88,3	97,6		575	81,1	49,8	77,0	85,2	
**Eggs (g/day)**												
	N	Mean	SD	CI 95%		anova p-value	N	Mean	SD	CI 95%		anova p-value
All	793	10,9	10,0	10,2	11,6		785	10,1	8,5	9,5	10,7	
**Age classes (years)**												
35-44	196	11,2	10,0	9,8	12,6	0,1783	191	10,6	9,1	9,3	11,9	0,1783
45-54	204	11,4	11,6	9,8	13,0		216	10,2	7,8	9,1	11,2	
55-64	219	10,9	9,5	9,6	12,2		214	9,9	9,3	8,7	11,2	
65-74	174	9,6	7,8	8,4	10,7		164	9,4	7,7	8,2	10,6	
**Italian Area**												
North	357	10,4	10,2	9,3	11,5	0,9016	359	10,7	9,7	9,7	11,7	0,9016
Center	180	10,8	9,5	9,4	12,2		184	10,0	8,1	8,9	11,2	
South	256	11,6	10,1	10,4	12,8		242	9,2	6,9	8,3	10,1	
**Education**												
Primary and middle school	201	11,4	11,7	9,8	13,0	0,6666	209	9,4	8,2	8,3	10,5	0,6666
High school and university	591	10,7	9,4	10,0	11,5		575	10,3	8,7	9,6	11,0	
**Milk (ml/day)**												
	N	Mean	SD	CI 95%		anova p-value	N	Mean	SD	CI 95%		anova p-value
**All**	**793**	**81,0**	**110,4**	**73,3**	**88,7**		**785**	**100,1**	**117,1**	**91,9**	**108,3**	
**Age classes (years)**												
35-44	196	71,3	99,3	57,4	85,2	0,6325	191	110,4	117,6	93,8	127,1	0,6325
45-54	204	73,0	105,5	58,5	87,5		216	108,0	126,0	91,2	124,8	
55-64	219	86,2	115,5	70,9	101,5		214	85,5	109,9	70,8	100,2	
65-74	174	100,2	123,6	81,8	118,5		164	93,0	109,5	76,3	109,8	
**Italian Area**												
North	357	81,6	110,1	70,2	93,0	0,4855	359	92,7	112,9	81,0	104,3	0,4855
Center	180	80,8	102,4	65,8	95,7		184	108,8	116,9	91,9	125,7	
South	256	80,2	116,6	65,9	94,5		242	104,6	123,1	89,1	120,1	
**Education**												
Primary and middle school	201	79,3	106,2	64,7	94,0	0,9280	209	101,2	120,8	84,9	117,6	0,9280
High school and university	591	81,5	112,0	72,4	90,5		575	99,9	116,0	90,5	109,4	

For meats intake, the following foods were considered: beef, veal, pork, horse, chicken, turkey, rabbit (domestic).
Dietary information was collected by the self-administered Italian version of the food frequency questionnaire (FFQ) of the European Prospective Investigation into Cancer (EPIC).
SD: standard deviation; CI: confidence interval.
Means and standard deviations were age standardized by European standard population distribution 2013 (except when they are reported by age-classes).
Anova to compare mean values among classes.
Pool of the following Italian regions: Piedmont, Lombardy, Liguria, Emilia Romagna, Tuscany, Lazio, Basilicata, Calabria, and Sicily.
Italian Area: North (Piedmont, Lombardy, Liguria, Emilia Romagna); Center (Tuscany, Lazio); South (Basilicata, Calabria, Sicily).

Table S.22. Age-standardized (European standard population) nutrients intake (EPIC questionnaire): proteins (total, animal and vegetable) mean by sex, age classes, geographical area and educational level.
Men and women residing in Italy aged 35-74 years, Health Examination Survey 2018-2019 - CUORE Project.

	Men						Women					
						Proteins Tot (% total Kcal)						
	N	Mean	SD	CI 95%		anova p-value	N	Mean	SD	CI 95%		anova p-value
**All**	**793**	**15,5**	**3,1**	**15,3**	**15,7**		**785**	**16,0**	**3,5**	**15,8**	**16,3**	
**Age classes (years)**												
35-44	196	15,8	2,6	15,5	16,2	0,9283	191	15,6	2,7	15,2	16,0	0,9283
45-54	204	15,3	2,7	15,0	15,7		216	16,2	3,7	15,7	16,7	
55-64	219	15,5	3,0	15,1	15,9		214	15,9	3,8	15,4	16,4	
65-74	174	15,3	4,3	14,6	15,9		164	16,5	4,0	15,9	17,1	
**Italian Area**												
North	357	15,3	2,6	15,0	15,6	0,0975	359	15,9	3,3	15,6	16,2	0,0975
Center	180	15,5	2,5	15,1	15,8		184	16,0	3,9	15,4	16,6	
South	256	15,8	4,0	15,3	16,3		242	16,3	3,7	15,8	16,7	
**Education**												
Primary and middle school	201	15,3	2,8	14,9	15,7	0,8686	209	16,2	3,4	15,8	16,7	0,8686
High school and university	591	15,6	3,2	15,3	15,8		575	16,0	3,6	15,7	16,3	
						Animal proteins (% total Kcal)						
	N	Mean	SD	CI 95%		anova p-value	N	Mean	SD	CI 95%		anova p-value
**All**	**793**	**10,4**	**3,7**	**10,1**	**10,6**		**785**	**10,8**	**4,1**	**10,5**	**11,1**	
**Age classes (years)**												
35-44	196	10,9	3,0	10,4	11,3	0,6278	191	10,6	3,1	10,1	11,0	0,6278
45-54	204	10,3	3,3	9,9	10,8		216	11,0	4,3	10,5	11,6	
55-64	219	10,2	3,5	9,7	10,7		214	10,6	4,4	10,1	11,2	
65-74	174	9,9	5,0	9,2	10,7		164	11,1	4,6	10,4	11,8	
**Italian Area**												
North	357	10,3	3,2	9,9	10,6	0,2704	359	10,8	3,8	10,4	11,2	0,2704
Center	180	10,1	3,1	9,7	10,6		184	10,7	4,5	10,0	11,3	
South	256	10,7	4,5	10,1	11,2		242	10,9	4,2	10,4	11,5	
**Education**												
Primary and middle school	201	10,3	3,2	9,9	10,8	0,3210	209	11,2	3,8	10,7	11,7	0,3210
High school and university	591	10,4	3,8	10,1	10,7		575	10,7	4,2	10,4	11,0	
						Vegetable proteins (% total Kcal)						
	N	Mean	SD	CI 95%		anova p-value	N	Mean	SD	CI 95%		anova p-value
**All**	**793**	**5,1**	**1,2**	**5,1**	**5,2**		**785**	**5,2**	**1,2**	**5,1**	**5,3**	
**Age classes (years)**												
35-44	196	5,0	1,1	4,8	5,1	<0,0001	191	5,1	1,1	4,9	5,2	<0,0001
45-54	204	5,0	1,1	4,8	5,2		216	5,2	1,3	5,0	5,3	
55-64	219	5,3	1,2	5,2	5,5		214	5,3	1,2	5,1	5,5	
65-74	174	5,3	1,4	5,1	5,5		164	5,4	1,4	5,2	5,6	
**Italian Area**												
North	357	5,1	1,1	4,9	5,2	0,0011	359	5,1	1,2	5,0	5,2	0,0011
Center	180	5,4	1,1	5,2	5,5		184	5,3	1,2	5,1	5,5	
South	256	5,1	1,3	4,9	5,3		242	5,3	1,3	5,2	5,5	
**Education**												
Primary and middle school	201	5,0	1,1	4,8	5,1	0,0060	209	5,0	1,3	4,9	5,2	0,0060
High school and university	591	5,2	1,2	5,1	5,3		575	5,3	1,2	5,2	5,4	

Dietary information was collected by the self-administered Italian version of the food frequency questionnaire (FFQ) of the European Prospective Investigation into Cancer (EPIC).
SD: standard deviation; CI: confidence interval.
Means and standard deviations were age standardized by European standard population distribution 2013 (except when they are reported by age-classes).
Anova to compare mean values among classes.
Pool of the following Italian regions: Piedmont, Lombardy, Liguria, Emilia Romagna, Tuscany, Lazio, Basilicata, Calabria, and Sicily.
Italian Area: North (Piedmont, Lombardy, Liguria, Emilia Romagna); Center (Tuscany, Lazio); South (Basilicata, Calabria, Sicily).

Table S.23. Age-standardized (European standard population) nutrients intake (EPIC questionnaire): lipids (animal and vegetable) and cholesterol mean by sex, age classes, geographical area and educational level. Men and women residing in Italy aged 35-74 years, Health Examination Survey 2018-2019 - CUORE Project.

	Men						Women					
	**Animal Lipids (% total Kcal)**											
	**N**	**Mean**	**SD**	**CI 95%**		**anova p-value**	**N**	**Mean**	**SD**	**CI 95%**		**anova p-value**
**All**	**793**	**17,9**	**5,3**	**17,6**	**18,3**		**785**	**17,6**	**5,1**	**17,2**	**18,0**	
**Age classes (years)**												
35-44	196	19,2	5,0	18,5	19,9	<0,0001	191	17,9	4,9	17,2	18,6	<0,0001
45-54	204	18,4	5,1	17,7	19,1		216	17,9	5,1	17,2	18,6	
55-64	219	17,2	5,2	16,5	17,9		214	17,3	5,4	16,6	18,0	
65-74	174	16,5	5,7	15,6	17,3		164	17,0	5,2	16,2	17,8	
**Italian Area**												
North	357	18,0	5,1	17,5	18,5	0,0074	359	17,7	4,9	17,2	18,2	0,0074
Center	180	17,2	5,2	16,4	18,0		184	16,9	5,1	16,1	17,6	
South	256	18,4	5,6	17,7	19,1		242	17,9	5,5	17,2	18,6	
**Education**												
Primary and middle school	201	18,2	5,3	17,4	18,9	0,0096	209	18,4	5,0	17,7	19,1	0,0096
High school and university	591	17,7	5,4	17,3	18,2		575	17,2	5,2	16,8	17,7	
	**Vegetable Lipids (% total Kcal)**											
	**N**	**Mean**	**SD**	**CI 95%**		**anova p-value**	**N**	**Mean**	**SD**	**CI 95%**		**anova p-value**
**All**	**793**	**17,1**	**5,7**	**16,7**	**17,5**		**785**	**19,9**	**6,3**	**19,5**	**20,4**	
**Age classes (years)**												
35-44	196	16,5	5,1	15,8	17,2	0,1321	191	19,5	5,7	18,7	20,4	0,1321
45-54	204	17,7	6,1	16,9	18,6		216	20,2	6,7	19,3	21,1	
55-64	219	17,0	5,8	16,2	17,8		214	19,9	6,2	19,0	20,7	
65-74	174	17,3	6,0	16,4	18,2		164	20,1	6,9	19,1	21,2	
**Italian Area**												
North	357	17,2	5,9	16,6	17,8	0,0056	359	20,2	6,7	19,5	20,8	0,0056
Center	180	18,1	5,9	17,2	18,9		184	20,2	6,4	19,3	21,1	
South	256	16,4	5,3	15,7	17,0		242	19,4	5,8	18,7	20,1	
**Education**												
Primary and middle school	201	16,0	5,7	15,2	16,7	<0,0001	209	18,8	5,8	18,0	19,6	<0,0001
High school and university	591	17,5	5,7	17,1	18,0		575	20,4	6,5	19,8	20,9	
	**Cholesterol (mg/day)**											
	**N**	**Mean**	**SD**	**CI 95%**		**anova p-value**	**N**	**Mean**	**SD**	**CI 95%**		**anova p-value**
**All**	**793**	**154,3**	**48,2**	**151,0**	**157,7**		**785**	**161,5**	**53,1**	**157,8**	**165,2**	
**Age classes (years)**												
35-44	196	160,7	44,2	154,5	166,8	0,2843	191	160,1	44,2	153,8	166,4	0,2843
45-54	204	156,9	43,2	150,9	162,8		216	162,7	50,4	156,0	169,4	
55-64	219	149,6	50,7	142,9	156,3		214	160,6	57,7	152,9	168,4	
65-74	174	148,2	56,2	139,9	156,6		164	162,5	61,0	153,1	171,8	
**Italian Area**												
North	357	150,0	43,3	145,5	154,5	0,0045	359	163,9	53,1	158,4	169,4	0,0045
Center	180	150,0	47,2	143,2	156,9		184	153,1	49,5	146,0	160,3	
South	256	162,0	55,9	155,2	168,9		242	164,2	56,2	157,1	171,3	
**Education**												
Primary and middle school	201	157,1	43,2	151,2	163,1	0,0153	209	169,6	56,2	162,0	177,2	0,0153
High school and university	591	153,5	49,8	149,5	157,5		575	158,7	51,4	154,5	162,9	

Dietary information was collected by the self-administered Italian version of the food frequency questionnaire (FFQ) of the European Prospective Investigation into Cancer (EPIC).
SD: standard deviation; CI: confidence interval.
Means and standard deviations were age standardized by European standard population distribution 2013 (except when they are reported by age-classes).
Anova to compare mean values among classes.
Pool of the following Italian regions: Piedmont, Lombardy, Liguria, Emilia Romagna, Tuscany, Lazio, Basilicata, Calabria, and Sicily.
Italian Area: North (Piedmont, Lombardy, Liguria, Emilia Romagna); Center (Tuscany, Lazio); South (Basilicata, Calabria, Sicily).

Table S.24. Age-standardized (European standard population) nutrients intake (EPIC questionnaire): saturated, polyunsaturated and monounsaturated fat, lipids total mean by sex, age classes, geographical area and educational level.

Men and women residing in Italy aged 35-74 years, Health Examination Survey 2018-2019 - CUORE Project.

	Men						Women					
	**Saturated fat (% total Kcal)**											
	N	Mean	SD	CI 95%		anova p-value	N	Mean	SD	CI 95%		anova p-value
**All**	**793**	**11,0**	**2,4**	**10,8**	**11,1**		**785**	**11,4**	**2,4**	**11,2**	**11,6**	
**Age classes (years)**												
35-44	196	11,3	2,3	11,0	11,6	<0,0001	191	11,6	2,3	11,2	11,9	<0,0001
45-54	204	11,3	2,3	10,9	11,6		216	11,5	2,3	11,2	11,8	
55-64	219	10,6	2,4	10,3	11,0		214	11,3	2,6	10,9	11,6	
65-74	174	10,4	2,7	10,0	10,8		164	11,1	2,5	10,7	11,5	
**Italian Area**												
North	357	11,2	2,4	10,9	11,4	0,0048	359	11,5	2,3	11,3	11,8	0,0048
Center	180	10,6	2,5	10,3	11,0		184	11,0	2,3	10,7	11,3	
South	256	10,9	2,5	10,6	11,2		242	11,5	2,7	11,1	11,8	
**Education**												
Primary and middle school	201	10,9	2,4	10,6	11,2	0,4720	209	11,6	2,4	11,3	11,9	0,4720
High school and university	591	11,0	2,5	10,8	11,2		575	11,3	2,4	11,1	11,5	
	**Polyunsaturated fat (% total Kcal)**											
	N	Mean	SD	CI 95%		anova p-value	N	Mean	SD	CI 95%		anova p-value
**All**	**793**	**5,0**	**1,3**	**4,9**	**5,1**		**785**	**5,3**	**1,4**	**5,2**	**5,4**	
**Age classes (years)**												
35-44	196	5,2	1,1	5,0	5,3	0,0069	191	5,3	1,2	5,1	5,4	0,0069
45-54	204	5,2	1,5	5,0	5,4		216	5,5	1,3	5,3	5,6	
55-64	219	4,8	1,3	4,7	5,0		214	5,3	1,3	5,1	5,4	
65-74	174	4,8	1,4	4,6	5,0		164	5,3	1,8	5,1	5,6	
**Italian Area**												
North	357	4,9	1,3	4,8	5,1	0,8683	359	5,4	1,5	5,3	5,6	0,8683
Center	180	5,1	1,2	4,9	5,3		184	5,2	1,2	5,0	5,4	
South	256	5,1	1,5	4,9	5,2		242	5,3	1,3	5,1	5,5	
**Education**												
Primary and middle school	201	5,0	1,4	4,8	5,2	0,9168	209	5,4	1,3	5,2	5,5	0,9168
High school and university	591	5,0	1,3	4,9	5,1		575	5,3	1,4	5,2	5,4	
	**Monounsaturated fat (% total Kcal)**											
	N	Mean	SD	CI 95%		anova p-value	N	Mean	SD	CI 95%		anova p-value
**All**	**793**	**16,1**	**3,7**	**15,9**	**16,4**		**785**	**17,6**	**4,0**	**17,4**	**17,9**	
**Age classes (years)**												
35-44	196	16,2	3,1	15,8	16,6	0,0473	191	17,6	3,4	17,1	18,0	0,0473
45-54	204	16,7	3,8	16,1	17,2		216	17,9	4,3	17,3	18,5	
55-64	219	15,8	3,9	15,3	16,4		214	17,5	3,8	17,0	18,0	
65-74	174	15,7	3,8	15,1	16,3		164	17,5	4,7	16,8	18,2	
**Italian Area**												
North	357	16,1	3,7	15,7	16,5	0,2615	359	17,7	4,2	17,3	18,1	0,2615
Center	180	16,5	3,6	16,0	17,1		184	17,7	3,8	17,2	18,2	
South	256	15,9	3,7	15,5	16,4		242	17,5	3,9	17,0	18,0	
**Education**												
Primary and middle school	201	15,4	3,6	14,9	15,9	0,0008	209	17,2	3,9	16,6	17,7	0,0008
High school and university	591	16,4	3,7	16,1	16,7		575	17,8	4,1	17,5	18,1	
	**Lipids Tot (% total Kcal)**											
	N	Mean	SD	CI 95%		anova p-value	N	Mean	SD	CI 95%		anova p-value
**All**	**793**	**35,1**	**6,6**	**34,6**	**35,5**		**785**	**37,5**	**6,5**	**37,1**	**38,0**	
**Age classes (years)**												
35-44	196	35,7	5,5	34,9	36,5	0,0009	191	37,5	5,7	36,7	38,3	0,0009
45-54	204	36,1	6,6	35,2	37,0		216	38,1	6,4	37,2	38,9	
55-64	219	34,2	6,9	33,3	35,1		214	37,2	6,4	36,3	38,1	
65-74	174	33,8	7,1	32,7	34,8		164	37,1	7,9	35,9	38,3	
**Italian Area**												
North	357	35,2	6,2	34,5	35,8	0,2901	359	37,9	6,6	37,2	38,6	0,2901
Center	180	35,3	6,4	34,3	36,2		184	37,0	5,9	36,2	37,9	
South	256	34,8	7,2	33,9	35,6		242	37,3	6,9	36,5	38,2	
**Education**												
Primary and middle school	201	34,1	6,6	33,2	35,0	0,0593	209	37,2	6,6	36,3	38,1	0,0593
High school and university	591	35,3	6,6	34,7	35,8		575	37,6	6,6	37,1	38,1	

Dietary information was collected by the self-administered Italian version of the food frequency questionnaire (FFQ) of the European Prospective Investigation into Cancer (EPIC).
SD: standard deviation; CI: confidence interval.
Means and standard deviations were age standardized by European standard population distribution 2013 (except when they are reported by age-classes).
Anova to compare mean values among classes.
Pool of the following Italian regions: Piedmont, Lombardy, Liguria, Emilia Romagna, Tuscany, Lazio, Basilicata, Calabria, and Sicily.
Italian Area: North (Piedmont, Lombardy, Liguria, Emilia Romagna); Center (Tuscany, Lazio); South (Basilicata, Calabria, Sicily)

Table S.25. Age-standardized (European standard population) nutrients intake (EPIC questionnaire) fiber, sodium, potassium, carbohydrate and simple carbohydrate mean by sex, age classes, geographical area and educational level.
Men and women residing in Italy aged 35-74 years, Health Examination Survey 2018-2019 - CUORE Project.

	Men						Women					
**Fiber (g/day)**												
	N	Mean	SD	CI 95%		anova p-value	N	Mean	SD	CI 95%		anova p-value
**All**	**793**	**9,9**	**2,7**	**9,7**	**10,0**		**785**	**11,3**	**3,0**	**11,1**	**11,5**	
**Age classes (years)**												
35-44	196	9,2	2,5	8,9	9,6	<0,0001	191	10,8	2,7	10,4	11,2	<0,0001
45-54	204	9,7	3,0	9,3	10,1		216	11,1	3,2	10,7	11,5	
55-64	219	10,2	2,5	9,9	10,5		214	11,7	3,0	11,3	12,1	
65-74	174	10,4	2,8	10,0	10,9		164	11,6	2,8	11,1	12,0	
**Italian Area**												
North	357	9,6	2,7	9,3	9,9	0,0049	359	11,1	3,1	10,8	11,4	0,0049
Center	180	10,4	2,8	10,0	10,8		184	11,5	2,9	11,1	12,0	
South	256	9,9	2,7	9,6	10,2		242	11,3	2,8	11,0	11,7	
**Education**												
Primary and middle school	201	9,5	2,4	9,2	9,8	0,0358	209	11,0	3,1	10,6	11,4	0,0358
High school and university	591	10,0	2,8	9,7	10,2		575	11,3	2,9	11,1	11,6	
**Sodium (g/day)**												
	N	Mean	SD	CI 95%		anova p-value	N	Mean	SD	CI 95%		anova p-value
**All**	**793**	**2,5**	**1,0**	**2,4**	**2,6**		**785**	**2,1**	**0,9**	**2,0**	**2,2**	
**Age classes (years)**												
35-44	196	2,7	1,1	2,5	2,8	<0,0001	191	2,3	1,0	2,2	2,4	<0,0001
45-54	204	2,6	1,0	2,5	2,7		216	2,2	0,9	2,1	2,3	
55-64	219	2,5	1,0	2,3	2,6		214	2,0	0,9	1,9	2,1	
65-74	174	2,2	0,9	2,0	2,3		164	1,8	0,9	1,6	1,9	
**Italian Area**												
North	357	2,5	1,0	2,4	2,6	0,9334	359	2,1	0,9	2,0	2,2	0,9334
Center	180	2,4	0,9	2,3	2,5		184	2,1	1,0	2,0	2,3	
South	256	2,5	1,1	2,4	2,7		242	2,0	1,0	1,9	2,2	
**Education**												
Primary and middle school	201	2,6	1,1	2,5	2,8	0,2252	209	1,9	0,9	1,8	2,0	0,2252
High school and university	591	2,5	1,0	2,4	2,6		575	2,2	1,0	2,1	2,2	
**Potassium (g/day)**												
	N	Mean	SD	CI 95%		anova p-value	N	Mean	SD	CI 95%		anova p-value
**All**	**793**	**3,2**	**1,0**	**3,1**	**3,3**		**785**	**3,0**	**1,0**	**3,0**	**3,1**	
**Age classes (years)**												
35-44	196	3,2	1,0	3,1	3,3	<0,0001	191	3,2	1,0	3,1	3,3	<0,0001
45-54	204	3,3	1,0	3,1	3,4		216	3,1	1,0	3,0	3,2	
55-64	219	3,2	1,1	3,1	3,3		214	2,9	1,0	2,8	3,1	
65-74	174	3,0	1,0	2,8	3,1		164	2,8	1,1	2,6	2,9	
**Italian Area**												
North	357	3,2	1,0	3,1	3,3	0,8018	359	3,0	1,0	2,9	3,1	0,8018
Center	180	3,1	0,9	3,0	3,3		184	3,1	1,0	3,0	3,2	
South	256	3,2	1,2	3,1	3,3		242	2,9	1,1	2,8	3,1	
**Education**												
Primary and middle school	201	3,2	1,1	3,0	3,3	0,0008	209	2,8	1,0	2,6	2,9	0,0008
High school and university	591	3,2	1,0	3,1	3,3		575	3,1	1,0	3,0	3,2	
**Carbohydrate (% total Kcal)**												
	N	Mean	SD	CI 95%		anova p-value	N	Mean	SD	CI 95%		anova p-value
**All**	**793**	**48,0**	**9,0**	**47,3**	**48,6**		**785**	**47,5**	**8,8**	**46,8**	**48,1**	
**Age classes (years)**												
35-44	196	47,3	7,8	46,2	48,4	0,2268	191	47,9	7,5	46,9	49,0	0,2268
45-54	204	47,3	8,9	46,0	48,5		216	46,9	8,6	45,7	48,0	
55-64	219	48,9	9,5	47,7	50,2		214	47,7	8,7	46,5	48,8	
65-74	174	48,5	9,9	47,0	50,0		164	47,4	11,1	45,7	49,1	
**Italian Area**												
North	357	47,5	8,4	46,6	48,4	0,0647	359	46,8	8,9	45,9	47,7	0,0647
Center	180	48,4	8,2	47,2	49,6		184	47,8	8,1	46,6	48,9	
South	256	48,3	10,1	47,0	49,5		242	48,1	9,2	47,0	49,3	
**Education**												
Primary and middle school	201	48,6	9,0	47,4	49,9	0,1040	209	48,0	8,7	46,8	49,1	0,1040
High school and university	591	47,7	8,9	47,0	48,4		575	47,3	8,9	46,5	48,0	
**Simple carbohydrates (% total Kcal)**												
	N	Mean	SD	CI 95%		anova p-value	N	Mean	SD	CI 95%		anova p-value
**All**	**793**	**18,6**	**6,2**	**18,1**	**19,0**		**785**	**20,2**	**6,6**	**19,7**	**20,6**	
**Age classes (years)**												
35-44	196	18,4	5,7	17,6	19,2	0,6066	191	20,9	6,4	20,0	21,8	0,6066
45-54	204	18,8	5,8	18,0	19,6		216	20,0	6,5	19,1	20,8	
55-64	219	18,4	6,4	17,5	19,2		214	20,2	6,3	19,3	21,0	
65-74	174	18,7	7,2	17,6	19,7		164	19,4	7,7	18,2	20,6	
**Italian Area**												
North	357	18,4	5,7	17,8	19,0	0,8063	359	20,5	6,8	19,8	21,2	0,8063
Center	180	18,5	5,6	17,7	19,3		184	20,0	6,4	19,1	20,9	
South	256	18,8	7,2	17,9	19,7		242	19,8	6,6	18,9	20,6	
**Education**												
Primary and middle school	201	19,4	7,1	18,5	20,4	0,0033	209	21,0	7,0	20,0	21,9	0,0033
High school and university	591	18,3	5,9	17,8	18,8		575	19,9	6,5	19,3	20,4	

Dietary information was collected by the self-administered Italian version of the food frequency questionnaire (FFQ) of the European Prospective Investigation into Cancer (EPIC).
SD: standard deviation; CI: confidence interval.
Means and standard deviations were age standardized by European standard population distribution 2013 (except when they are reported by age-classes).
Anova to compare mean values among classes.
Pool of the following Italian regions: Piedmont, Lombardy, Liguria, Emilia Romagna, Tuscany, Lazio, Basilicata, Calabria, and Sicily.
Italian Area: North (Piedmont, Lombardy, Liguria, Emilia Romagna); Center (Tuscany, Lazio); South (Basilicata, Calabria, Sicily).
